# *Syringa oblata* genome provides new insights into molecular mechanism of flower color differences among individuals and biosynthesis of its flower volatiles

**DOI:** 10.3389/fpls.2022.1078677

**Published:** 2022-12-21

**Authors:** Lifei Chen, Bin Xia, Ziwei Li, Xiaowei Liu, Yun Bai, Yujia Yang, Wenjie Gao, Qingran Meng, Ning Xu, Ying Sun, Qiang Li, Liran Yue, Miao He, Yunwei Zhou

**Affiliations:** ^1^ College of Horticulture, Jilin Agricultural University, Changchun, China; ^2^ College of Landscape Architecture, Northeast Forestry University, Harbin, China; ^3^ School of Ecological Technology and Engineering, Shanghai Institute of Technology University, Shanghai, China; ^4^ School of Perfume and Aroma Technology, Shanghai Institute of Technology University, Shanghai, China; ^5^ School of Forestry, Northeast Forestry University, Harbin, China

**Keywords:** genome assembly, flower color formation, individuals, volatiles biosynthesis, *Syringa oblata*

## Abstract

*Syringa oblata* is a high ornamental value tree owing to its elegant colors, unique aromas and wide adaptability, however, studies on the molecular mechanism underlying the formation of its ornamental traits are still lacking. Here, we presented a chromosome-scale genome assembly of *S. oblata* and the final genome size was 1.11 Gb with a contig N50 of 4.75 Mb, anchored on 23 chromosomes and was a better reference for *S. oblata* transcriptome assembly. Further by integrating transcriptomic and metabolic data, it was concluded that *F3H, F3’H, 4CL* and *PAL*, especially the *F3’H*, were important candidates involved in the formation of floral color differences among *S. oblata* individuals. Genome-wide identification and analysis revealed that the TPS-b subfamily was the most abundant subfamily of TPS family in *S. oblata*, which together with the CYP76 family genes determined the formation of the major floral volatiles of *S. oblata*. Overall, our results provide an important reference for mechanistic studies on the main ornamental traits and molecular breeding in *S. oblata*.

## Introduction

*Syringa oblata* is among the high ornamental value trees in the middle latitudes of Eurasia and North America and it has been cultivated in China for more than 1,000 years owing to its elegant colors, unique aromas and wide adaptability (Li et al., 2006). In previous studies, the majority of research on *S. oblata* has focused on the determination of volatile components, identification of active substances and transcriptomics. For example, previous studies of transcriptomic analysis in *S. oblata* revealed that molecular mechanism of the accumulation of flavonoids in response to different light intensity. cDNA libraries from three inflorescence developmental stages were sequenced and assembled to obtain differentially expressed genes involved in floral pigment biosynthesis and fragrance metabolism in *S. oblate*. (Zheng et al., 2015; Cui et al., 2019; Liu et al., 2019; Gegen et al., 2022; Tai et al., 2022). Despite the high economic and ornamental values of *S. oblata*, its genetic and breeding research still lag behind other flowering plants.

*S. oblata* flowers usually show varying degrees of purple color on different individuals (Zhang, 2011). The delphinidin-3-O-rutinoside and cyanidin-3-O-rutinoside are the main anthocyanins currently reported for *S. oblata* petal coloration (Zhang, 2011; Ma et al., 2022), and their syntheses are involved in the phenylpropanoid biosynthesis and anthocyanin biosynthesis pathways. The precursor of anthocyanin biosynthesis, Phenylalanine, forms anthocyanins after a series of catalysis by enzymes such as phenylalanine lyase (PAL), 4-coumarate–CoA ligase (4CL), etc., followed by the formation of glycosidic bonds under the action of glucosyltransferase and conversion to stable anthocyanins (Holton and Cornish, 1995; Zhang et al., 2014). These genes have been reported to be intimately associated with the formation of purple or blue flowers in plants. For example, naringenin 3-dioxygenase (*F3H*) mutant lines of *Petunia* obtained *via* Cas9-ribonucleoproteins delivery exhibit a paler purple (Yu et al., 2021), flower-specific expression of the *Phalaenopsis* flavonoid 3′, 5′-hydoxylase (*F3’5’H*) modifies flower color pigmentation in *Petunia* and *Lilium (* Qi et al., 2013 *)*. Previous studies on the color of *S. oblata* flowers have elucidated some transcription factors associated with petal color changes during flower development (Ma et al., 2022), however, the molecular mechanism underlying the formation of differences in floral color among *S. oblata* individuals is still lacking, which is of great significance for its molecular breeding of flower color.

*S. oblata* has a strong floral fragrance, and previous studies have revealed that the main volatiles of its flowers are terpenoids such as lilac aldehyde, lilac alcohol, α-pinene, sabinene and β-pinene (Li et al., 2006; Gegen et al., 2022; Wang et al., 2022), while terpene synthase (TPS) is the pivotal gene family for the biosynthesis of plant terpenes (Chen et al., 2011). Plant secondary metabolites usually require post-modification processes to produce biologically active metabolites (Li et al, 2009) and many *S. oblata* flower volatiles undergo post-modification processes, such as lilac aldehyde and lilac alcohol, which are oxygenated derivatives of linalool formed through the catalysis of cytochrome P450 (Raguso, 2016). Former studies on the volatiles of *S. oblata* have mainly focused on the determination of composition (Yao-zu et al., 1987; Li et al., 2006; Wu et al., 2021; Gegen et al., 2022), while little attention has been paid to the molecular mechanism of their synthesis and post-modification processes. Therefore, genome-wide identification and analysis of the TPS and post-modification genes of the volatiles of *S. oblata* are important references for revealing the molecular mechanism of its volatile biosynthesis.

As genome sequencing, assembly and annotation methods continue to evolve and progress, new plant reference genomes are constantly being published (Sun et al., 2022). In our study, we reported a chromosome-scale *S. oblata* genome with a longer contig and it was a better reference for its transcriptome assembly. Meanwhile, The combination of the metabolic results and transcriptomic data of *S. oblata* individuals with different colors were used to screen the key genes regulating the flower color. In addition, *S. oblata* TPS and related post-modification genes were genome-wide identified and analyzed to elucidate the molecular mechanism of its volatile biosynthesis. The genome sequence analysis, transcriptome and metabolic data from this study are significant references for studying the mechanism of important ornamental traits and breeding in *S. oblata*.

## Materials and methods

### Plant material and genome sequencing

The sequenced *S. oblata* was maintained at the Harbin Institute of Landscape Architecture and Greening Science in good growing condition, and its young leaves were collected for whole-genome and Hi-C sequencing. On the same day of the flowering period, petals at the expanding flower bud stage were collected from *S. oblata* individuals (Sob1, Sob2, Sob3) of different flower colors for RNA-seq, and their petal colors were compared using NCS color cards during the blooming period, and petals were also collected for anthocyanin determination. Plants were immediately stored in liquid nitrogen until they were shipped back to the laboratory and stored at -80°C.

Genomic DNA was extracted from leaves of *S. oblata* using the DNAsecure Plant Kit (TIANGEN, China). DNA sequencing libraries were constructed based on the Illumina library preparation protocols. Paired-end (PE150) library with insert size of 350 bp was constructed according to the manufacturer’s instructions and sequencing using the Illumina Hiseq X-ten (Illumina, USA). Then the raw reads were filtered out the adapter sequences and the low-quality and duplicated reads to obtain clean reads. For Pacbio libraries, SMRT Bell libraries with an insert size of 60 kb were constructed and then sequenced on the PacBio Sequel platform (Pacific Biosciences, USA) using the P6 polymerase/C4 chemistry combination, based on the manufacturer’s procedure. A Hi-C library was generated following the approach described by Lieberman-Aiden et al. (Lieberman-Aiden et al., 2009). Briefly, purified DNA was obtained by formaldehyde fixation of chromatin, cell lysis, Hind III nuclease digestion, recovery, ligation and protein removal by protease. The purified DNA was sheared into 350-bp fragments and ligated to adaptors. The fragments labeled with biotin were extracted using streptavidin beads and after PCR enrichment, the libraries were sequenced on Illumina HiSeq X instrument (Illumina, USA).

### Genome survey and assembly

Iteratively selected 17 bp base sequences (K-value=17) were used for K-mer analysis. Kmer frequency distributions were counted and K-mer depth distribution curves were calculated and then GenomeScope (Vurture et al., 2017) was used to assess genome size, percentage of repetitive sequences, and heterozygosity ratio.

*De novo* assembly of the long reads from the PacBio SMRT Sequencer was performed using wtdbg2 (Luo et al., 2012; Md et al., 2019). The Fuzzy Bruijn Graph algorithm was used to assemble and integrate 1,024bp sequences from the reads into vertex sequences, and then based on the position of the vertex sequences on the reads, the vertex sequences were concatenated to obtain the genome sequences. Subsequently, the Hi-C sequencing data were aligned to the assembled scaffolds by BWA-mem (Md et al., 2019) and the scaffolds were clustered onto chromosomes with LACHESIS (Burton et al., 2013). A BUSCO analysis was conducted to determine genome completeness using BUSCO (Simão et al., 2015) together with the embryophyta odb10 database.

### RNA-seq and assembly

Total RNA was extracted using RNAprep Pure Plant Kit (TIANGEN, China). The library insert size was 300 bp and more than 6 Gb of 150-bp pair-end clean reads were obtained using the Illumina Hiseq novoseq 6000 platform (Illumina, USA). Transcriptome public data of *S. oblata* was downloaded from SRA database. Trinity (Grabherr et al., 2011) was used to assemble the transcriptome without reference and HISAT2 (Kim et al., 2019) was used to assemble the transcriptome with reference, selecting the default parameters.

### Genome annotation

A combined strategy based on homology comparison and ab initio search was used for repeat sequence annotation. Tandem repeats were extracted by ab initio prediction using TRF (Benson, 1999). The homolog prediction used Repbase database (Jurka et al., 2005) employing RepeatMasker (Chen, 2004) to extracted repeat regions. And ab initio prediction built *de novo* repetitive elements database by LTR_FINDER (Xu and Wang, 2007), RepeatScout (Price et al., 2005), and RepeatModeler2 (Flynn et al., 2020), then was supplied to RepeatMasker for DNA-level repeat identification.

Gene structural annotation was based on ab initio prediction, homology-based prediction and RNA-Seq assisted prediction. For Ab initio predication, Augustus (Stanke and Morgenstern, 2005) AND GlimmerHMM (Majoros et al., 2004) were used. Proteins sequences of Antirrhinum majus, Arabidopsis thaliana, Fraxinus excelsior, Olea europaea, Sesamum indicum and Solanum tuberosum were aligned using TblastN (E-value ≤1e-5) (Camacho et al., 2009) with the genome of *S. oblata* and then the matching proteins were aligned to the homologous genome sequences for accurate spliced alignments with GeneWise (Madeira et al., 2022). Transcriptome reads assemblies were generated with Trinity for the genome annotation. The non-redundant reference gene set was generated by merging genes predicted by three methods with EvidenceModeler (Haas et al., 2008). The protein-coding genes functions were assigned using BLAST against public protein databases, including NR (https://ftp.ncbi.nlm.nih.gov/blast/db/FASTA/), SwissProt (Boeckmann et al., 2003), KEGG (Kanehisa and Goto, 2000), InterPro (Jones et al., 2014), GO (Ashburner et al., 2000) and Pfam (Finn et al., 2013).

The tRNAs were predicted using tRNAscan-SE (Lowe and Eddy, 1997). The rRNAs were predicted by using BLAST against rRNA sequences of relative species. Other ncRNAs, including miRNAs, snRNAs were identified by searching against the Rfam (Griffiths-Jones et al., 2003) using the infernal (Nawrocki and Eddy, 2013).

### Comparative genomic and phylogenetic analysis

Orthologous relationships between genes of *S. oblata* and 13 other species including *Oryza sativa* (Yu et al., 2002), *O. europaea* (Unver et al., 2017), *F. excelsior* (Sollars et al., 2017), *Vitis vinifera* (Jaillon et al., 2007), *S. indicum* (Wang et al., 2014), *A. majus* (Li et al., 2019), *A. thaliana* (The Arabidopsis Genome, 2000), *Brassica napus* (Chalhoub et al., 2014), *Populus trichocarpa* (Tuskan et al., 2006), *Solanum lycopersicum* (Sato et al., 2012), *P. axillaris, P. inflata* (Bombarely et al., 2016) and *S. tuberosum* (Xu et al., 2011) were inferred through all-against-all protein sequence similarity searches with OthoMCL (Li et al., 2003) and retained only the longest predicted transcript per locus. Single-copy gene families were subjected to MUSCLE (Edgar, 2004) multiple sequence alignment and ambiguously aligned positions were trimmed using Gblocks (Dereeper et al., 2008) and the tree was inferred using RAxML (Stamatakis, 2014). Divergence time estimation was performed using MCMCtree (http://abacus.gene.ucl.ac.uk/software/paml.html) in the PAML package (Yang, 1997) with time correction points for *P. trichocarpa – A. thaliana* (97- 109 Mya), *P. trichocarpa – V. vinifera* (107-135 Mya), *A. majus – A. thaliana* (111-131 Mya), and *B. napus – O. sativa* (148-173 Mya), respectively. The likelihood model in the software package Cafe´ (De Bie et al., 2006) was used to perform gene families expansion and contraction analysis.

### Genome synteny and whole-genome duplication analysis

BLASTP (E-value ≤ 1e-5) analysis of proteins was performed between and within Jasminum sambac (Xu et al., 2022), *S. oblata* and *O. europaea* and *V. vinifera*, and then based on the location of genes and blast results using MCscanX (Wang et al., 2012), searching for co-linear regions within or between genomes. The Ks values of ortholog pairs or paralog pairs were plotted using the WGDI (Sun et al., 2021), and plotted the frequency distribution of Ks versus gene pairs. Synteny dot plots were plotted by dot-plotter in McscanX.

### Identification of anthocyanin biosynthesis genes, TPS genes and CYP450 genes

Genes potentially involved in anthocyanin biosynthesis (ko00940, ko00942) were identified in *S. oblata* and other representative plants based on the KAAS web service in KEGG (Moriya et al., 2007). The PF01397 and PF03936 files were used to Hmmsearch for *S. oblata* and other related species protein data, respectively (P<10.0), and the two datasets were combined and de-redundant to obtain the Hmmsearch dataset (Gbenro et al., 2020). To make the dataset more complete, a blastp search of the S. oblata and other related species protein libraries was performed using the *A. thaliana* TPS protein sequences (Aubourg et al., 2002) to obtain the homologous sequence dataset, which was further combined and de-redundant with the Hmmsearch dataset, and the dataset was structurally verified using the Pfam to obtain structurally complete TPS genes. Hmmsearch (PF00067) and homology comparisons based on *A. thaliana* CYP450 proteins (Paquette et al., 2000) were used to identify CYP450 gene family members of *S. oblata*, and further Pfam was used to verify the structural completeness of the identification results. TBtools was used to Chromosome localization and mapping (Chen et al., 2020).

### Determination of *S. oblata* flower anthocyanin and volatiles

Samples for the determination of anthocyanins from *S. oblata* petals were prepared with reference to the method in Zhang’s published paper (Zhang, 2011). The chromatographic conditions were as follows: the chromatographic instrument was a WATERS ACQUITY UPLC (Waters, USA), the column was BEH C18 (2.1X150mm 1.7um), the mobile phase was acetonitrile (A) - 0.1% formic acid (B), gradient elution, the flow rate was 0.3ml/min, the column temperature was 45°C, the injection volume was 5ul, the wavelength was 200-500nm, the mass spectrometer was WATERS MALDI SYNAPT Q-TOF MS (Waters, USA), ion source was ESI, ion scan mode was ESI+, scan range was 20-2000 m/z, detection voltage was 1800 V, Desolvation Gas Flow: 700 lit/hr, Cone Gas Flow (L/Hr): 50 lit/hr. The standard curve was prepared using anthocyanin standards for quantitative analysis.

The quick-frozen flowers were ground to a powder in liquid nitrogen and 1.0 g was weighed into a headspace flask and held at 40°C for 30 min for equilibration. Fifteen microliters of 2-octanol (CAS: 123-96-6) was added as an internal standard. Headspace extraction was performed using divinylbenzene/carboxy/polydimethylsiloxane fibers (MilliporeSigma, USA) for 30 min, followed by desorption at 250°C for 3 min using a Model 7890A-5975C equipped with an HP-INNOWAX column (Agilent, USA) with nitrogen as the carrier gas at a flow rate of 3 ml/min without splitting. The temperature setting of the GC was: from 40°C to 120°C at a rate of 4°C/min1; to 180°C at a rate of 2°C/min1; then to 260°C at a rate of 20°C and held for 10 minutes. Scans were performed from m/z 35 to 550 in electron impact mode at 70 eV. All analyses were performed in triplicate.

## Result

### Genome sequencing, assembly and annotation

The genome size of *S. oblata* was estimated to be 1,232.83 Mb with heterozygosity of 1.32% and a repeat content of 66.56% on the basis of k-mer statistics (**Supplementary Table 1** and **Supplementary Figure 1**). In total, 146.00 Gb of PacBio long reads (~118.43X coverage of the genome) and 53.00 Gb of Illumina clean reads (~42.99X coverage of the genome) were generated (**Supplementary Table 2**). The total length of the final assembly was 1,112.64 Mb with 2,417 contigs and a contig N50 of 4.75 Mb (**Table 1**). The genome size was close to the results based on genome surveys. The assembly was further improved with Hi-C data, of the total scaffold sequences, 98.60% was anchored to 23 chromosome-level pseudomolecules (**Supplementary Table 3** and **Supplementary Figure 2**). The assessment of *S. oblata* genome completeness with BUSCO indicated that 92.00% of the 1,440 BUSCO groups were present in the genome assembly (**Supplementary Table 4**), and CEGMA results indicated 96.37% coverage of the conserved core eukaryotic genes (**Supplementary Table 5**). Additionally, 98.75% of the Illumina short reads were mapped to the genome assembly, and 97.93% of genome was covered by reads (**Supplementary Table 6**), further supporting a high base accuracy of the *S. oblata* genome assembly.

**Table:** Table 1 The major characteristics of *S. oblata* genome.

Parameter	Number/Size
Estimated genome size (Gb)	1.23
Assembled genome size (Gb)	1.11
Chromosome-anchored scaffolds (Gb)	1.10
Karyotype (chromosomes, 2n)	46
N50 of contigs (Mb)	4.75
Number of contigs	2,417
Longest contigs (Mb)	21.49
N50 of scaffolds (Mp)	44.71
Longest scaffolds (Mb)	70.97
GC content (%)	34.67
Repeat sequences (%)	56.64
Number of protein-coding genes	42,531
Number of non-coding RNAs	6,427

**Figure 1:**
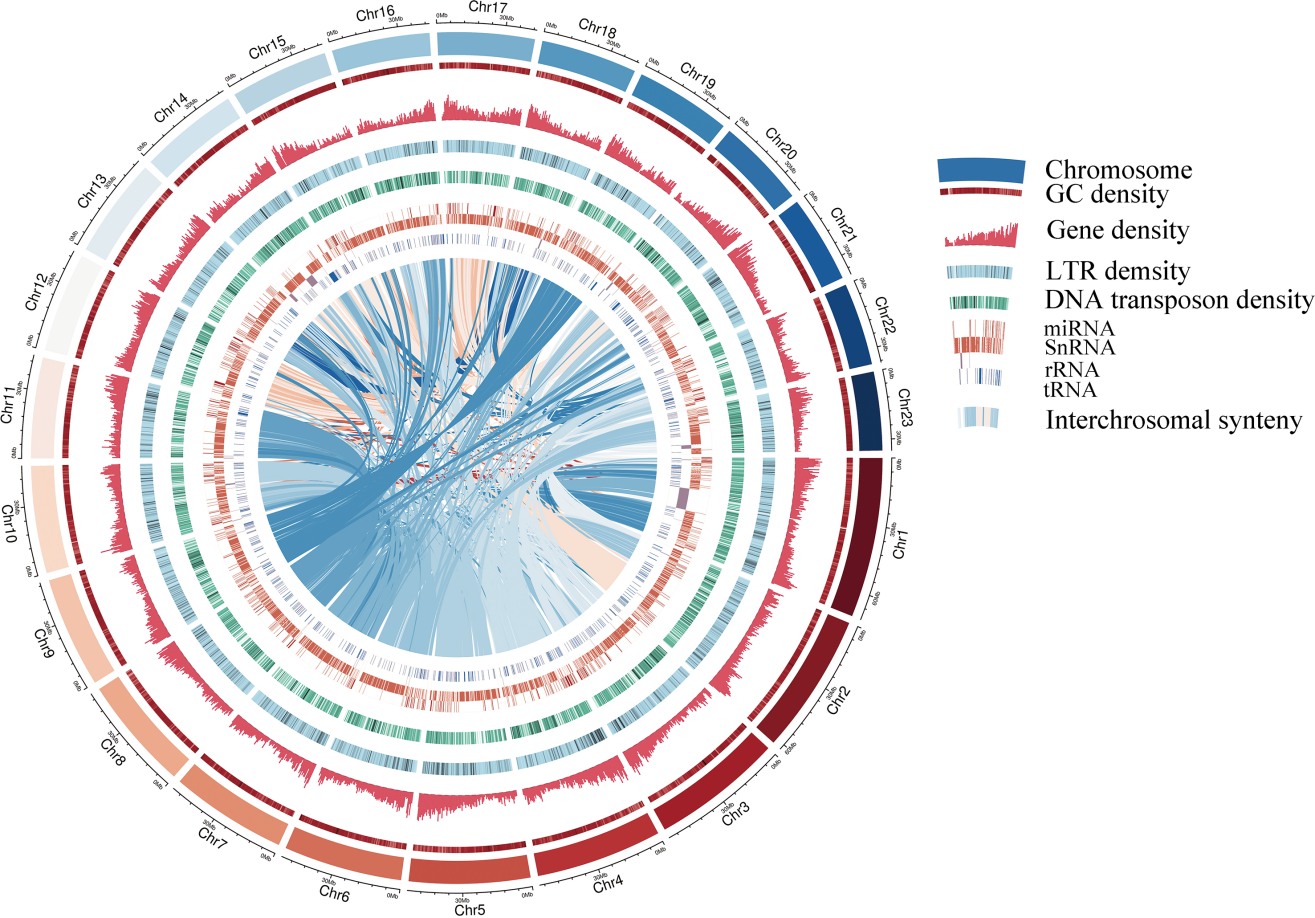
Circular diagram showing the characteristics of the *S. oblata* genome Gene density, LTR (Long terminal repeat transposon) density and DNA transposon density are 500kb window size.

A pipeline combining *de novo* predictions, homology-based predictions, and RNA sequencing data were used to construct gene models for the S. oblata genome. A total of 42,531 genes were annotated this way, with an average length of 4,103 bp and an average coding sequence length of 1,082 bp, similar to those of other reported plants (**Supplementary Table 7** and **Supplementary Table 8**). The resulting protein models were then compared with protein sequences in six protein databases; NR, SwissProt, KEGG, InterPro, GO and Pfam. We found that 40,621 (95.50%) gene products could be annotated by at least one of the databases (**Supplementary Table 9**). The assembled draft S. oblata genome contained 56.64% repetitive sequences. Long terminal repeats (LTRs) of retroelements were the most abundant interspersed repeat, occupying 52.07% of the genome, followed by Tandem repeats at 5.30% (**Supplementary Table 10**). Genes annotated as encoding non-coding RNAs (ncRNAs) in the current genome included 873 microRNAs (miRNAs), 732 transfer RNAs (tRNAs), 295 ribosomal RNAs (rRNAs), and 4,527 small nuclear RNAs (snRNAs) (**Supplementary Table 11**). These results further supported the completeness of *S. oblata* genome sequence and a schematic representation of the genome was given in **Figure 1**.

Compared with other published *S. oblata* genomes, our genome had a higher Contig N50 with its longest contig reaching 21.49 Mb, which was superior to other *S. oblata* genomes (**Supplementary Table 12**). To further validate the quality of our genome assembly and annotation, the published *S. oblata* transcriptome data were assembled with our and other published genomes as reference genome. The results showed that the transcriptome assembled with our genome as the reference genome had a higher alignment rate, especially a higher aligned one-time rate (**Supplementary Table 13**).

### Comparative genomic and phylogenomic analyses

**Figure 2:**
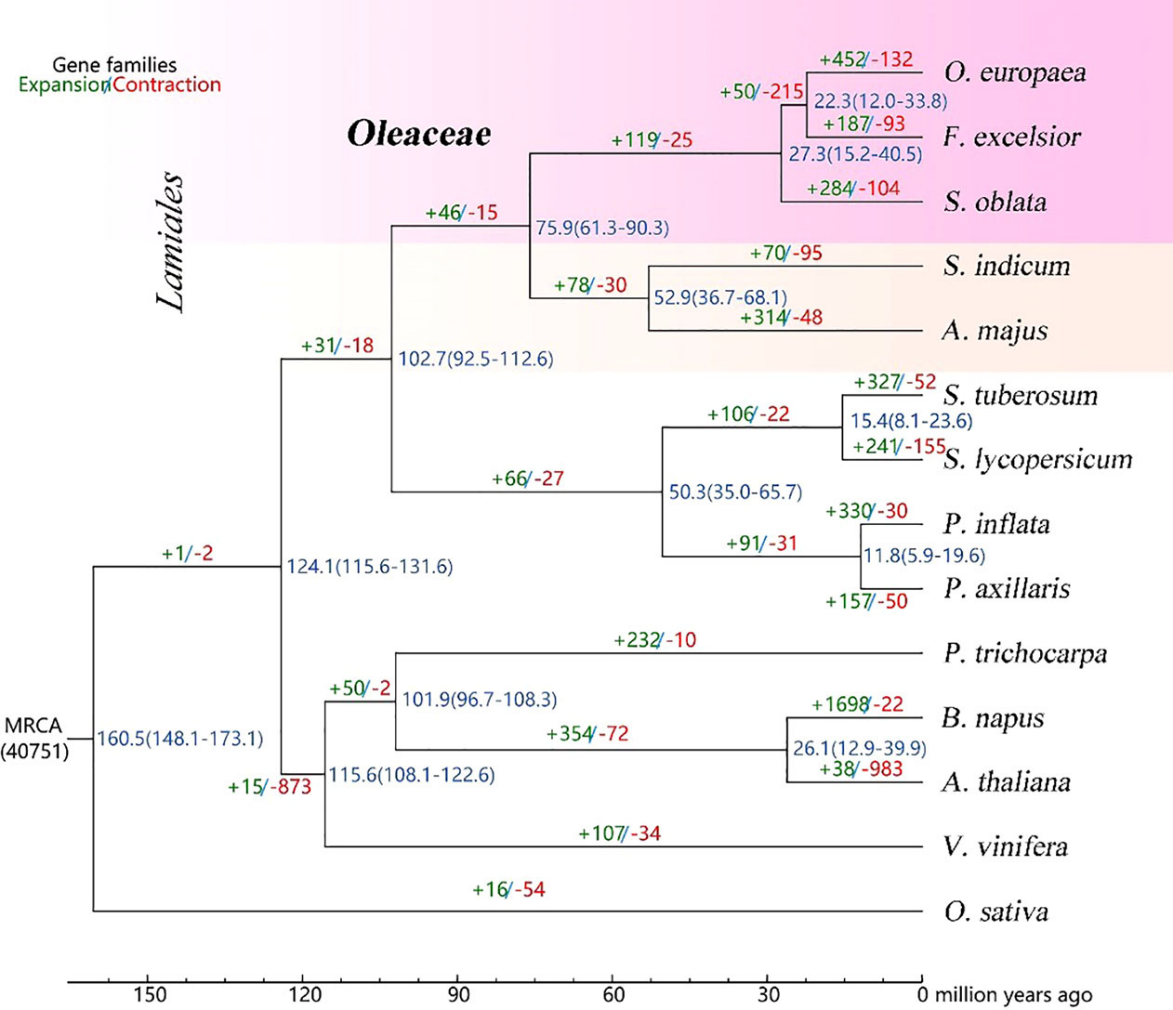
Phylogenetic tree and estimated the divergence times Blue numbers on the nodes are divergence time to present (in Mya).

The gene families were analyzed for the genomes of *S. oblata* and 13 other representative species. Based on the analysis of gene family clustering, a total of 40,768 gene families were identified, of which 7,023 were shared by all 14 species, and 41 of these shared families were single-copy gene families (**Supplementary Figure 3**). Further, phylogenetic tree and estimating divergence times analysis were performed based on 615 single-copy genes. (**Figure 2**). Phylogenomic analysis showed that *S. oblata* was most related to the ancestor of *O. europaea* and *F. excelsior*, with an estimated divergence time of 27.3 million years ago (MYA). The expanded and contracted gene families of the 14 plant species were compared with their most recent common ancestor (MRCA), *S. oblata* has gained more gene families (284) than it lost (104), among these genes. KEGG studies based on the 284 expanded gene families showed enrichment of genes including those encoding Phenylpropanoid biosynthesis, Sesquiterpenoid and triterpenoid biosynthesis and Biosynthesis of secondary metabolites were enriched (**Supplementary Table 14**), suggesting the secondary metabolism of S. oblata was enhanced in evolution. Patterns of gene-family sharing among Lamiales species were shown in **Supplementary Figure 4** for 16,525 families, including 33,022 proteins, of which 338 families were S. oblata-specific, containing 3,635 proteins. Interestingly, KEGG analyses found these S. oblata-specific genes were particularly enriched in the terms Phenylpropanoid biosynthesis, Sesquiterpenoid and triterpenoid biosynthesis, Degradation of aromatic compounds (**Supplementary Table 15**).

**Figure 3:**
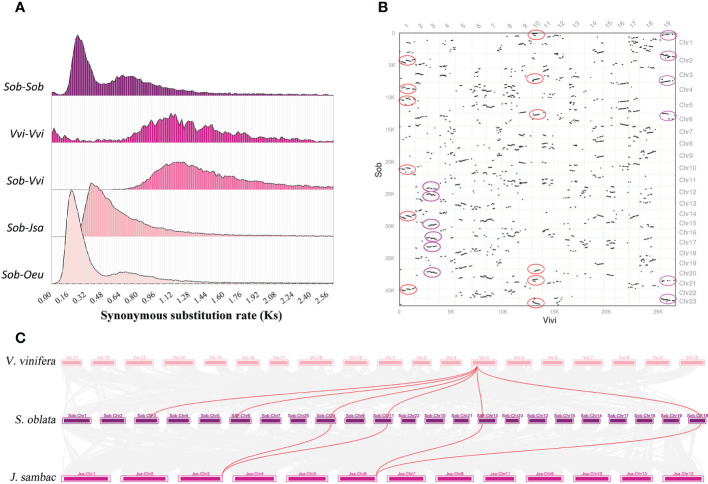
The genome-wide duplication events of *S. oblata*. **(A)** Ks density profiles of *S. oblata, V. vinifera* and *S. oblata* versus *J. sambac, V. vinifera* and *O. europaea*. (Sob : S. oblata,Vvi : V. vinifera, Jsa : J. sambac, Oeu : O. europaea) **(B)** Pairwise synteny visualization of *V. vinifera* versus *S. oblata*. **(C)** Karyotype map of the genomes of *S. oblata, J. sambac* and *V. vinifera*.

To resolve the genome-wide duplication events of *S. oblata*, three Oleaceae species (*J. sambac*, *S. oblata* and *O. europaea*) and *V. vinifera* were selected for comparative genomic analysis. Based on the calculated Ks distribution, two characteristic peaks appeared in the Ks distribution of *S. oblata*, which was smaller than the Ks peaks of *V. vinifera* and *S. oblata* versus *V. vinifera*, indicating that two independent genome-wide duplication events occurred in *S. oblata* after divergence from *V. vinifera* (**Figure 3A**). By comparing the Ks value profiles of *S. oblata* and *S. oblata* versus *J. sambac*, *O. europaea*, it was concluded that the first whole-genome duplication event of *S. oblata* occurred before divergence from *J. sambac* and the other shared with *O. europaea* occurred after divergence from *J. sambac*. Further analysis of homologous gene synteny between *S. oblata* and *V. vinifera*, *S. oblata* and *J. sambac* revealed six homologous blocks were found in some regions of the *V. vinifera* genome and a 2:1 relationship between *S. oblata* and *J. sambac*. (**Figures 3B**, **C**). In summary, after divergence from *V. vinifera*, *S. oblata* and *J. sambac* shared a common whole-genome triplication (WGT) event before divergence, and after divergence from *J. sambac*, *S. oblata* and *O. europaea* shared a whole-genome duplication (WGD) event.

### Analysis of molecular mechanism of floral color differences among *S. oblata* individuals

**Figure 4:**
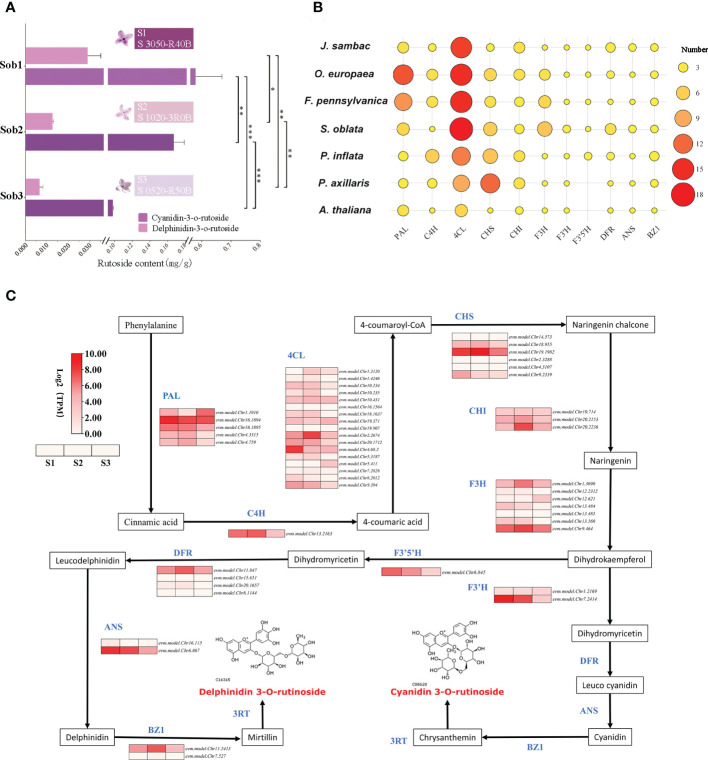
Analysis of molecular mechanism of floral color differences among *S. oblata* individuals. **(A)** The content of delphinidin-3-O-rutinoside and cyanidin-3-O-rutinoside of different *S. oblata* individuals. **(B)** Heat map of the number of delphinidin-3-O-rutinoside and cyanidin-3-O-rutinoside biosynthesis-related genes in different species. **(C)** The biosynthesis pathway and the expression profile for delphinidin-3-O-rutinoside and cyanidin-3-O-rutinoside in *S. oblata* individuals. T test (* : P ≤ 0.05, ** : P ≤ 0.01, *** :P ≤ 0.001) was used for statistical analyses; mean ± sd.

In our study, the contents of delphinidin-3-O-rutinoside and cyanidin-3-O-rutinoside in the petals of *S. oblata* individuals were quantified and their contents were found to be significantly and positively correlated with the purple-deepening (**Figure 4A**). By homology matching, 50 genes from 8 gene families involved in the biosynthesis of cyanidin-3-O-rutinoside and delphinidin-3-O-rutinoside were annotated and four gene families (*4CL, F3H, F3’H, DFR*) were increased in S. oblata compared to other species (**Figure 4B**; **Supplementary Table 16**). By analyzing the co-expression patterns of each synthase genes of *S. oblata* individuals, nine candidate genes affecting flower color differences among individuals of *S. oblata* were predicted (**Figure 4C**; **Supplementary Table 16**, higher expression in S1 than in S2, S3). Further correlation analysis of the contents of delphinidin-3-O-rutinoside and cyanidin-3-O-rutinoside with the expression pattern of synthase genes resulted in *evm.model.Chr13.484 (F3H), evm.model.Chr7.2414 (F3’H), evm.model.Chr8.2012 (4CL)* and *evm.model.Chr16.1894 (PAL)* showed significant (p < 0.05) positive correlations with their contents (**Supplementary Figure 5**). In addition, the expression pattern of synthase was analyzed by clustering analysis, and *evm.model.Chr7.2414 (F3’H)* showed the greatest distance from all other genes and the greatest variation among different individuals (**Supplementary Figure 6**). The above results suggested that *F3H, F3’H, 4CL* and *PAL*, especially the *F3’H*, were important candidates for regulating flower color differences among *S. oblata* individuals.

### Analysis of molecular mechanism of biosynthesis of *S. oblata* flower volatiles

**Figure 5:**
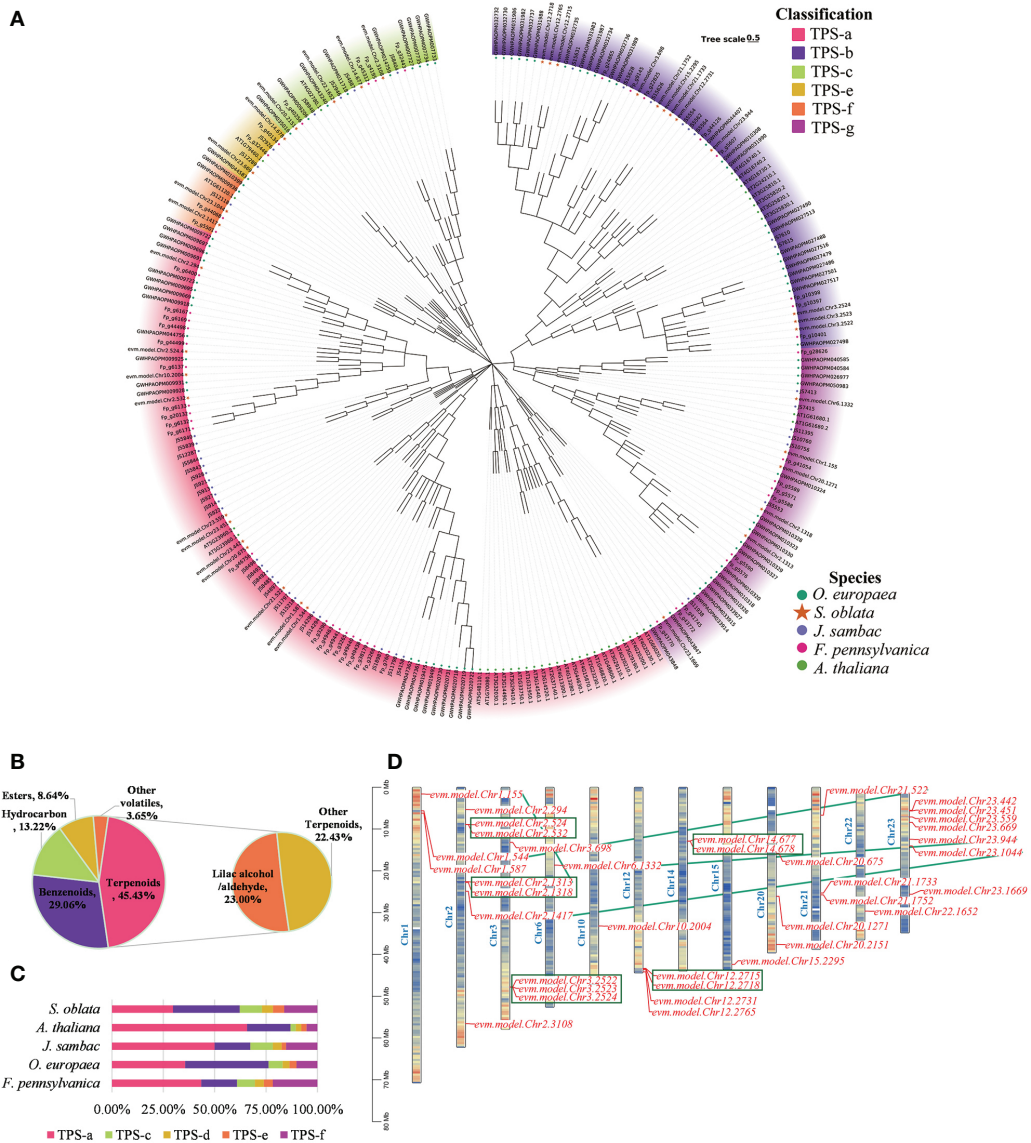
Analysis of molecular mechanism of biosynthesis of *S. oblata* flower volatiles. **(A)** Evolutionary trees of A. thaliana, J. sambac, O. europaea and F. pennsylvanica TPS genes. **(B)** The volatile components of S. oblata. **(C)** Percentage of TPS subgroups in different species. **(D)** Chromosome location map of TPS genes in *S. oblata*.

The flower of *S. oblata* had a fragrant aroma. In this study, the volatile components of *S. oblata* were determined and terpenes were found to be the most abundant volatiles (**Figure 5B**). Based on homologous sequence comparison and Pfam search, 37 TPS genes in S. oblata were identified, and evolutionary trees were further constructed with TPS genes of *A. thaliana, J. sambac, O. europaea* and *F. pennsylvanica* (**Figure 5A**; **Supplementary Table 17**). TPS genes of *S. oblata* were classified into six subgroups, TPS-a, TPS-b, TPS-c, TPS-e, TPS-f and TPS-g. The TPS-b subgroup had the highest percentage of genes of total TPS genes in S. oblata compared to other species, accounting for 32.43% (*A. thaliana*: 21.05%, *J. sambac*: 17.39%, *O. europaea*: 32.00%, and *F. pennsylvanica*: 17.39%) (**Figure 5C**). Nineteen *S. oblata* TPS genes (51.35% of all genes) underwent duplication, 10 of these genes underwent recent tandem duplication events, forming five gene clusters (green box markers) on chromosomes 2, 3, 12 and 14, and 10 genes underwent chromosomal duplication events, with tandem and chromosomal duplication resulting in TPS genes expansion in the S. oblata genome (**Figure 5D**).

The most abundant of the *S. oblata* terpene volatiles were Lilac aldehyde and lilac alcohol (**Figure 5B**), which were derivatives of the monoterpene linalool. Linalool was catalyzed by successive oxygenation of P450 superfamily genes to Lilac aldehyde and lilac alcohol. A total of 287 P450 genes were identified in S. oblata genome, and by further constructing an evolutionary tree with the TPS genes of *A. thaliana*, the P450 genes of S. oblata were classified into 9 classes and 50 families (**Supplementary Figure 7**; **Supplementary Table 18**). Genes of the CYP76 family were important genes for catalyzing the formation of Lilac aldehyde and lilac alcohol from linalool, and 14 CYP76 family members, of which 8 genes underwent tandem duplication and formed three gene clusters on chromosomes 4,10 and 12 (red gene markers), and tandem duplication resulted in the amplification of *S. oblata* CYP76 family members (**Supplementary Figure 8**).

## Discussion

In view of the importance of *S. oblata* in horticulture and the need for further research, the genome of S. oblata will provide an important reference for elucidating the molecular mechanisms of its major ornamental traits and molecular breeding. Our study, we sequenced, assembled and annotated the genome of *S. oblata*. The combination of Second- and Third-generation sequencing and Hi-C technology has shown high efficiency in assembling complex plant genomes (Kersey, 2019). The genome of S. oblata has high heterozygosity (1.32%) and highly repetitive sequences (56.64%). The *S. oblata* genome was assembled in our study by combining the Illumina short reads and the PacBio long reads sequenced with a contig N50 of 4.75 Mb and a largest contig of 21.49Mb. Its contig N50 was better than those genomes of *O. europaea, F. excelsior* and *O. fragrans* and other published *S. oblata* genomes. Further, those sequences were scaffolded into 23 chromosomes using Hi-C. The reference-based transcriptome assembly strategy requires a reference genome and the quality of the genome will directly influence the accuracy of the assembled transcripts (Martin and Wang, 2011). Compared with other published *S. oblata* genomes, the transcriptome assembly using our genome as reference possessed a higher alignment rate, indicating that our genome was a better reference for assembling *S. oblata* transcriptome.

Whole-genome duplication provides species with abundant evolutionary material that enhances species diversity and environmental adaptability (Sémon and Wolfe, 2007). Recent study has shown that an recent whole-genome triplication event occurred in J. sambac (Xu et al., 2022). In our study, we identified that the S. oblate has experienced one WGT event and one WGD event after the WGT-γ using Ks value profiles and synteny analysis, and among them, one was shared with J. sambac (WGT) and another occurred divergence with J. sambac (WGD).

The flowers of *S. oblata* had an elegant purple color and anthocyanins mainly contribute to its coloration, while delphinidin-3-O-rutinoside and cyanidin-3-O-rutinoside were the most abundant anthocyanins reported in *S. oblata* currently (Zhang, 2011; Ma et al., 2022). In this study, by transcriptome sequencing and quantitative analysis of anthocyanins in the petals of different S. oblata individuals, *F3H, F3’H, 4CL* and *PAL* were screened as important candidates for the regulation of anthocyanin contents, of which *PAL* and *4CL* acted as key rate-limiting enzymes in the phenylpropane biosynthesis pathway and functioned in flower or fruit color formation in many species, such as the different deletion mutation in the *PAL* gene affected the peel color of *Mangifera indica* (Zhao et al., 2022), *4CL* transcript levels affected metabolite flux of anthocyanin in *Narcissus tazetta* Flower (Yang et al., 2021), while F3H has been reported to regulate the purple color of petals in Petunia (Yu et al., 2021). Notably, further clustering analysis showed that the expression pattern of F3’H differed particularly in different *S. oblata* individuals, and *F3’H* played an influential role in regulating purple traits in flowers or fruits of different individuals of the same species, such as the red mutant of the dark purple petunia Ipomoea tricolor lacked the *F3’H* gene (Hoshino et al., 2003), and the increased dose of the recessive allele in the F3’H gene shifted the major anthocyanin in the storage roots of *Ipomoea batatas* from purplish cyanidin derivatives to reddish pelargonidin derivatives (Tanaka et al., 2019). As a result, we speculated that the differences in flower color among *S. oblata* individuals was regulated by a combination of *F3H, F3’H, 4CL* and *PAL* genes, emphatically the *F3’H* gene may play a key role.

Depending on the structure, plant floral volatiles can be divided into three main groups: terpenoids, phenylpropanoids/benzenoids and fatty acids (Muhlemann et al., 2014), and previous studies indicated that terpenes were the main components of *S. oblata* volatiles (Li et al., 2006; Gegen et al., 2022; Wang et al., 2022). Lilac aldehyde and lilac alcohol were the main volatiles of the S. oblata flowers found in our study which were oxygenated derivatives of the monoterpenoid linalool (Kreck et al., 2003; Boachon et al., 2015). Whereas the TPS-b subfamily is responsible for the synthesis of monoterpenes in angiosperms (Jia et al., 2022), members of the CYP76 family were shown to be involved in the sequential oxygenation-catalyzed biosynthesis of linalool to lilac aldehyde and lilac alcohol (Boachon et al., 2015). Yan et al. collected and analyzed a new monoterpene synthase gene, named *SoLIM*, which was classified as TPS-b subfamily member from the flowers of *Syringa oblata* and *S. oblata* var. *alba* to regulate D-limonene emissions during the flowering phase (Yan et al., 2019). Some cytochrome P450 genes were reported to be involved in metabolism of terpenoid volatiles, such as homologous with *V. vinifera* cytochrome P45076A2 and CYP76A2 in Syringa oblate (Zheng et al., 2015). In our study, the *S. oblata* TPS-b subfamily had the most members and its CYP76 family genes were amplified by tandem repeats forming gene clusters. Additional studies have shown that lilac aldehyde and lilac alcohol can enhance the attractiveness of plants to pollinating insects (Plepys et al., 2002; Dötterl et al., 2007; Jürgens et al., 2014), so the amplification of TPS-b subfamily and CYP76 family genes of *S. oblata* may improve competitiveness to attract insect pollination.

In summary, we published a chromosome-scale S. oblata genome with a longer contig and it was a better reference for transcriptome assembly. Genome-wide annotation and transcriptomic and metabolic data integration improved our understanding of the molecular mechanism underlying the formation of floral color differences among *S. oblata* individuals. In addition, the genome-wide identification and analysis of TPS and CYP450 genes in volatiles of *S. oblata* provided a basis for the elucidation of the mechanism of volatile compounds biosynthesis mechanism and further research. These results are important references for the resolution of molecular mechanism of horticultural traits and molecular breeding in *S. oblata*.

## Data availability statement

The original contributions presented in the study are publicly available. This data can be found here: CNGBdb(https://db.cngb.org/), CNP0003698.

## Author contributions

YZ, MH, BX, and LC conceived and designed the study. ZL and YY prepared the materials. BX, ZL, YY, WG, QM, and XL performed data analyses. LC, BX, YB, YS, QL, and LY wrote the manuscript. All authors contributed to the article and approved the submitted version.
