# Supplementary material for: Syringa oblata genome provides new insights into molecular mechanism of flower color differences among individuals and biosynthesis of its flower volatiles
Source: Front Plant Sci. 2022 Dec 21;13:1078677. doi: 10.3389/fpls.2022.1078677 (PMC9811319; doi:10.3389/fpls.2022.1078677)
Supplement: Supplementary file 1 [file DataSheet_1.docx]

Supplementary Material

# Supplementary Figures and Tables

## Supplementary Figures


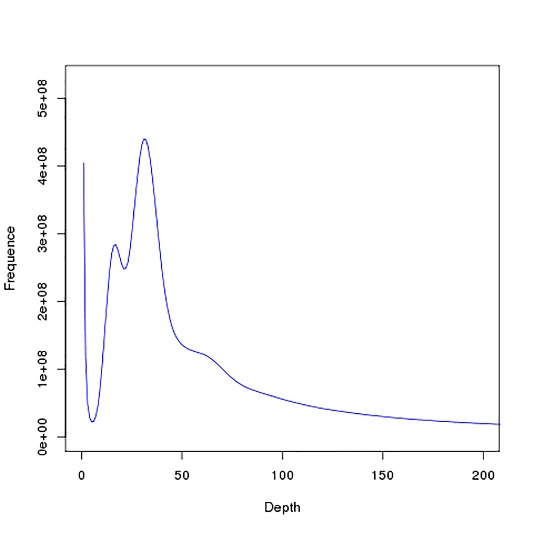


**Supplementary Figure 1.** K-mer analysis for estimating the genome size of *S. oblata.*


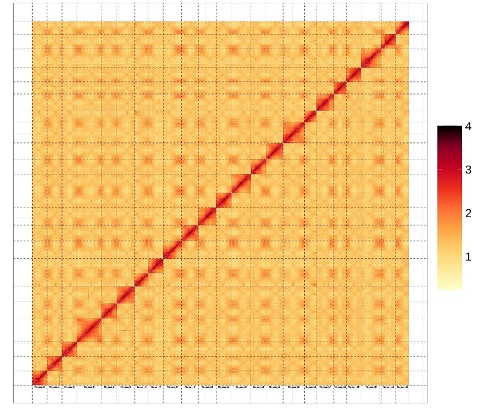


**Supplementary Figure 2.** Hi-C map of the *S. oblata* genome showing genome-wide all-by-all interactions.


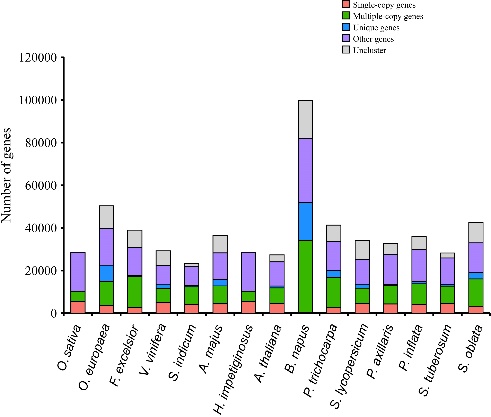


**Supplementary Figure 3.** The distribution of genes in different species


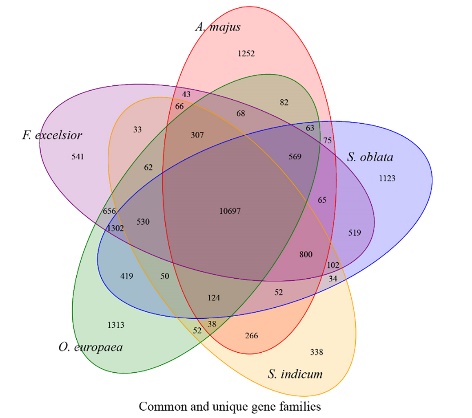


**Supplementary Figure 4.** Venn diagram of genes sharing by five *Lamiales* species


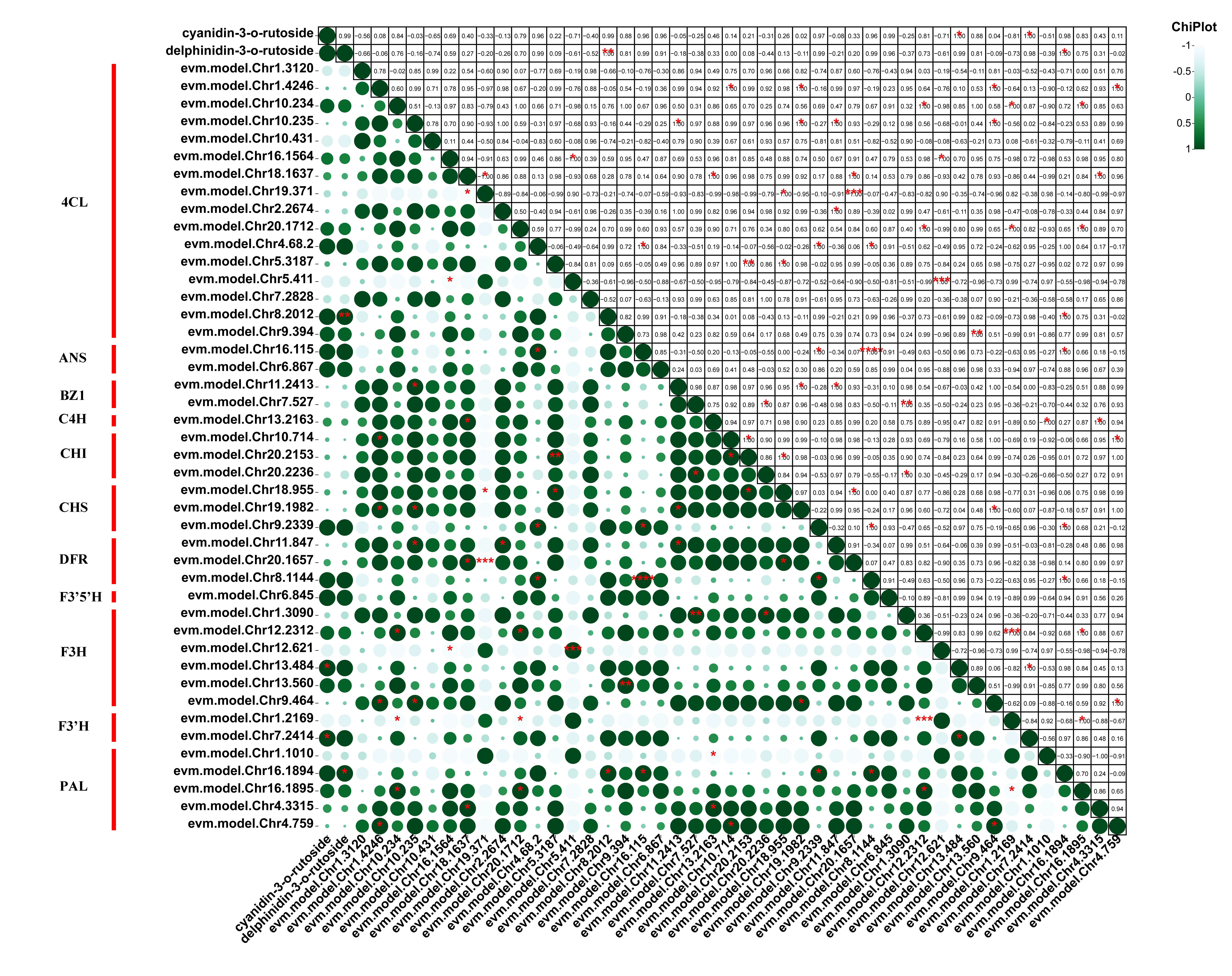


**Supplementary Figure 5.** Correlation of anthocyanin synthesis-related genes with the contents of delphinidin-3-O-rutinoside and cyanidin-3-O-rutinoside


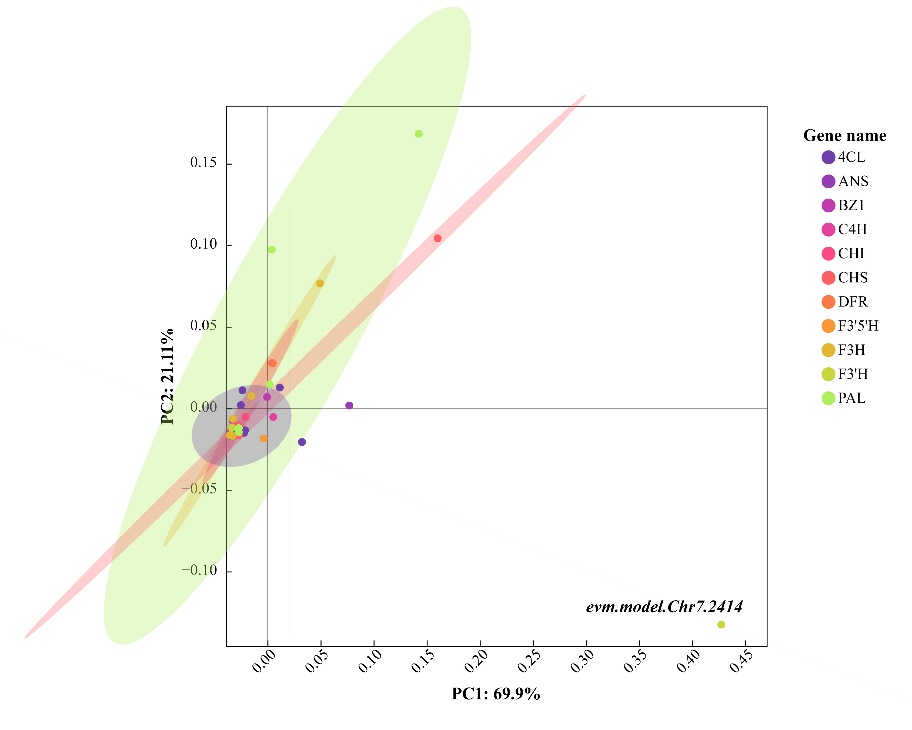


**Supplementary Figure 6.** Clustering analysis of expression patterns of anthocyanin synthesis-related genes in *S. oblata* individuals


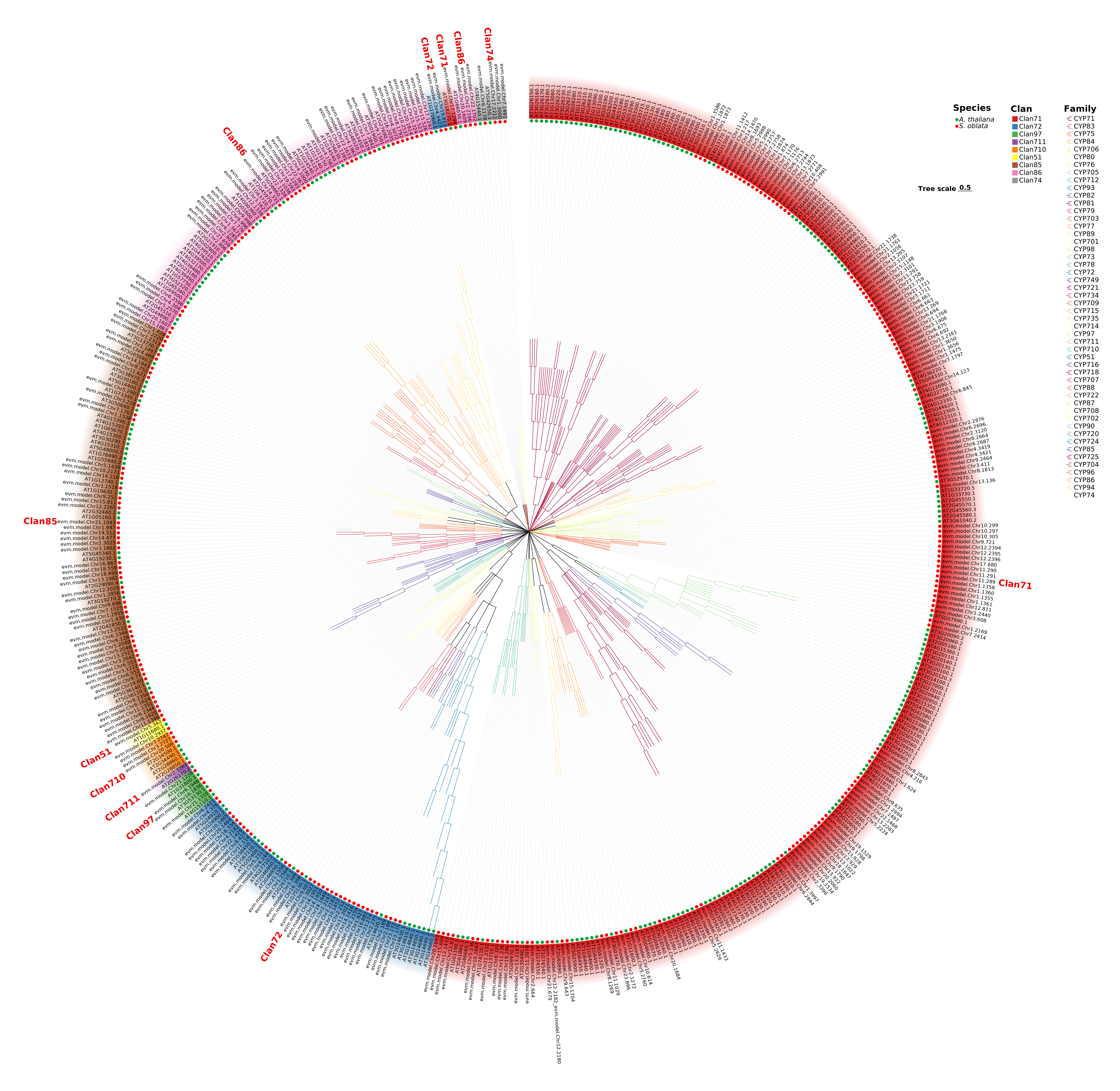


**Supplementary Figure 7.** Evolutionary tree of the CYP450 superfamily of *S. oblata* and *A. thaliana.*

**
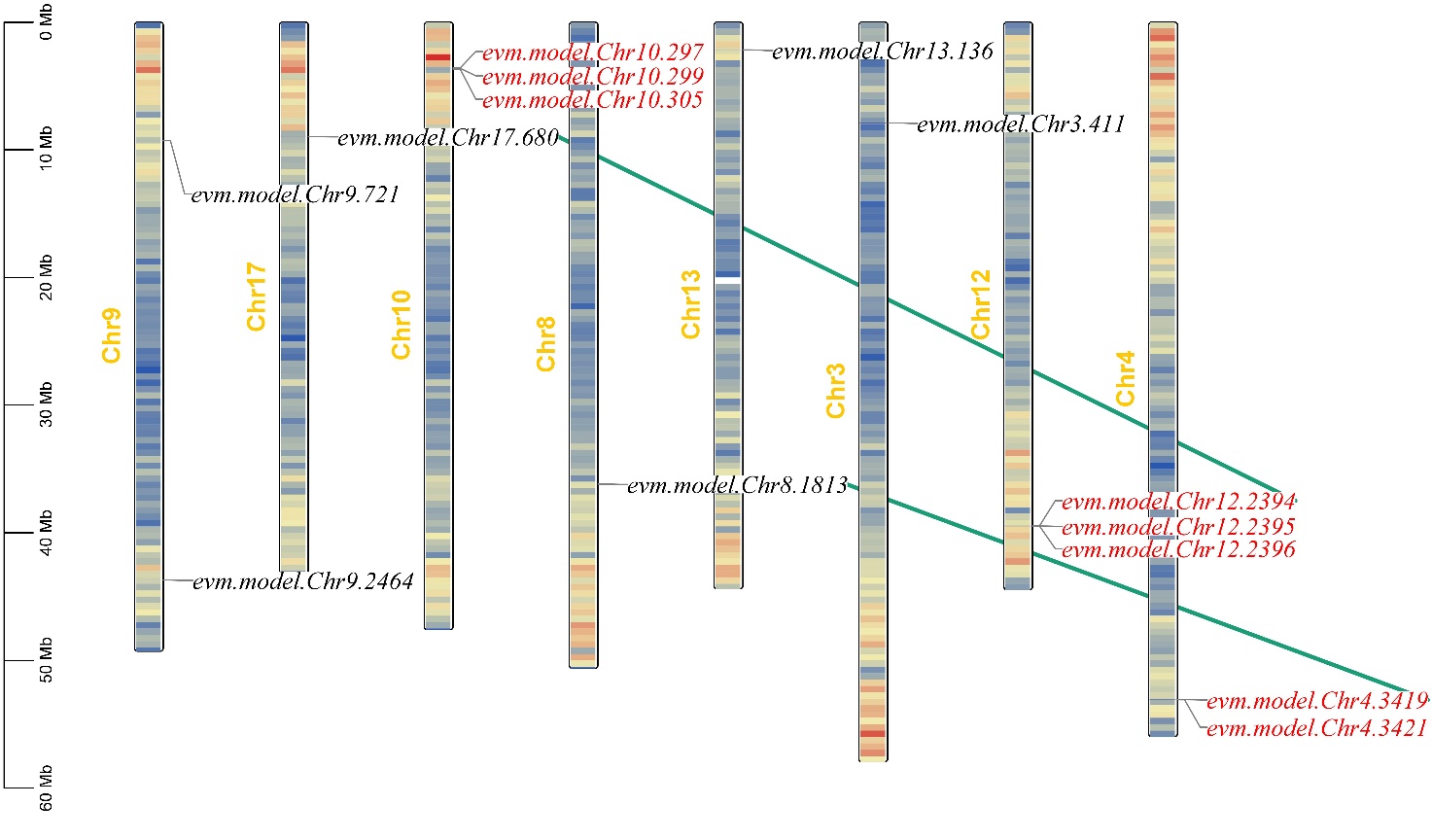
**

**Supplementary Figure 8.** Chromosomal localization of CYP76 gene family in *S. oblata.*

## Supplementary tables

**Supplementary Table 1.** Survey statistic results of *S. oblata*

| Kmer | Depth | n_kmer | Genome_size(M) | Revised Genome_size(M) | Heterozygous_rate(%) | Repeat_rate(%) |
| --- | --- | --- | --- | --- | --- | --- |
| 17 | 30 | 37389538217 | 1246.32 | 1232.83 | 1.32 | 66.56 |

**Supplementary Table 2.** Sequencing data statistics of *S. oblata*

| Pair-end libraries | Insert size | Total data (G) | Read length (bp) | Sequence coverage (X) |
| --- | --- | --- | --- | --- |
| Illumina reads | 350bp | 53 | 150 | 42.99 |
| PacBio reads | -- | 146 | -- | 118.43 |
| Total | -- | 199 | -- | 161.42 |

**Supplementary Table 3.** Genome mount rate of *S. oblata*

| Class | Scaffold Number | Total Length |
| --- | --- | --- |
| place | 23 | 1097260998 |
| unplace | 657 | 15596632 |
| total | 680 | 1112857630 |
| mount rate | 0.986 | |

**Supplementary Table 4.** Assessment the gene coverage rate using BUSCO

| Species | BUSCO notation assessment results |
| --- | --- |
| *S. oblata* | C:92.0%[S:78.5%,D:13.5%],F:1.7%,M:6.3%,n:1440 |
| C：Complete BUSCOs | |
| S：Complete and single-copy BUSCOs | |
| D：Complete Duplicated BUSCOs | |
| F：Fragmented BUSCOs | |
| M：Missing BUSCOs | |
| n：Total BUSCO groups searched | |

**Supplementary Table 5.** Assessment the gene coverage rate using CEGMA

| Species | Complete | | Complete + Partial | |
| --- | --- | --- | --- | --- |
|  | # Prots | %Completeness | # Prots | %Completeness |
| *S. oblata* | 234 | 94.35 | 239 | 96.37 |

**Supplementary Table 6.** Coverage statistics of *S. oblata* genome

|  |  | % of Percentage |
| --- | --- | --- |
| Reads | Mapping rate (%) | 98.75 |
| Genome | Average sequencing depth | 34.34 |
|  | Coverage (%) | 97.93 |
|  | Coverage at least 4X (%) | 97.31 |
|  | Coverage at least 10X (%) | 95.4 |
|  | Coverage at least 20X (%) | 78.95 |
| Average sequence depth: The average depth of each base on the genome that is covered by reads; Coverage:  The proportion of genomes that were covered by reads. | | |

**Supplementary Table 7.** Basic statistical results of gene structure prediction of *S. oblata* genome

| Gene set | | Number | Average transcript length(bp) | Average CDS length(bp) | Average exons per gene | Average exon length(bp) | Average intron length(bp) |
| --- | --- | --- | --- | --- | --- | --- | --- |
| De novo | Augustus | 60534 | 2929.17 | 899.47 | 3.9 | 230.46 | 699.2 |
|  | GlimmerHMM | 115921 | 8276.3 | 548.25 | 2.94 | 186.29 | 3977.39 |
|  | SNAP | 48643 | 19353.63 | 511.7 | 4.17 | 122.76 | 5946.92 |
|  | Geneid | 95404 | 4948.28 | 660.21 | 3.75 | 176 | 1558.65 |
|  | Genscan | 65294 | 10475.16 | 982.61 | 5.27 | 186.6 | 2225.23 |
| Homolog | *S. tuberosum* | 40878 | 3101.61 | 1257.87 | 3.63 | 346.07 | 699.79 |
|  | *S. indicum* | 38091 | 3380.9 | 1240.36 | 3.81 | 325.81 | 762.57 |
|  | *A. thaliana* | 75219 | 1793.14 | 784.61 | 2.61 | 300.43 | 625.8 |
|  | *O. europaea var sylvestris* | 45117 | 2563.62 | 1084.71 | 3.21 | 338.03 | 669.52 |
|  | *A. majus* | 45611 | 2782.61 | 1142.38 | 3.32 | 343.99 | 706.69 |
|  | *F. excelsior* | 58129 | 2689.78 | 903.22 | 3.5 | 257.88 | 713.93 |
| RNAseq | PASA | 45137 | 3643.37 | 906 | 4.62 | 196.17 | 756.5 |
|  | Transcripts | 77535 | 4574.31 | 1023.87 | 3.76 | 272.62 | 1288.4 |
| EVM |  | 64625 | 3268.8 | 882.97 | 3.94 | 224.05 | 811.22 |
| Pasa-update |  | 64485 | 3248.09 | 883.42 | 3.93 | 224.59 | 806.1 |
| Final set |  | 42531 | 4103.59 | 1082.88 | 4.78 | 226.77 | 800.12 |

**Supplementary Table 8.** Basic statistical results of gene structure prediction of *S. oblata* and relative species

| Species | Number | Average transcript length(bp) | Average CDS length(bp) | Average exons per gene | Average exon length(bp) | Average intron length(bp) |
| --- | --- | --- | --- | --- | --- | --- |
| *S. oblata* | 42531 | 4103.59 | 1082.88 | 4.78 | 226.77 | 800.12 |
| *A. majus* | 37234 | 2570.75 | 1038.22 | 4.22 | 245.82 | 475.42 |
| *F. excelsior* | 50841 | 4169.29 | 1196.92 | 5.57 | 214.73 | 649.82 |
| *O. europaea var sylvestris* | 60214 | 2512.58 | 1005.58 | 4.2 | 239.43 | 470.95 |
| *A. thaliana* | 48359 | 2056.72 | 1296.77 | 5.92 | 219 | 154.42 |
| *S. tuberosum* | 28333 | 4183.62 | 1282.2 | 5.14 | 249.62 | 701.4 |
| *S. indicum* | 23988 | 3371.34 | 1344.09 | 5.6 | 239.86 | 440.35 |

**Supplementary Table 9.** The statistical results of gene function annotation of *S. oblata* genome

|  | Number | Percent(%) |
| --- | --- | --- |
| Total | 42531 | - |
| Swissprot | 32026 | 75.3 |
| Nr | 40329 | 94.8 |
| KEGG | 32743 | 77 |
| InterPro | 38311 | 90.1 |
| GO | 22648 | 53.3 |
| Pfam | 31029 | 73 |
| Annotated | 40621 | 95.5 |
| Unannotated | 1910 | 4.5 |

**Supplementary Table 10.** Summary of Repeat contents in *S. oblata* genome

| Type | Length(bp) | % of Genome |
| --- | --- | --- |
| Tandem repeats | 59013301 | 5.3 |
| DNA | 15464192 | 1.39 |
| LINE | 3726973 | 0.33 |
| SINE | 2328 | 0 |
| LTR | 579449751 | 52.07 |
| Unknown | 27926268 | 2.51 |
| Total | 613468319 | 55.13 |

**Supplementary Table 11.** The statistical results of non-coding RNA of *S. oblata*

| Type | | Copy number | Average length(bp) | Total length(bp) | % of genome |
| --- | --- | --- | --- | --- | --- |
| miRNA | | 873 | 115.32 | 100676 | 0.009047 |
| tRNA | | 732 | 74.86 | 54797 | 0.004924 |
| rRNA | rRNA | 295 | 203.63 | 60071 | 0.005398 |
|  | 18S | 37 | 747.49 | 27657 | 0.002485 |
|  | 28S | 67 | 135.45 | 9075 | 0.000815 |
|  | 5.8S | 23 | 124.3 | 2859 | 0.000257 |
|  | 5S | 168 | 121.9 | 20480 | 0.00184 |
| snRNA | snRNA | 4527 | 108.92 | 493086 | 0.044308 |
|  | CD-box | 4300 | 107.37 | 461687 | 0.041487 |
|  | HACA-box | 71 | 130.01 | 9231 | 0.000829 |
|  | splicing | 152 | 140.74 | 21393 | 0.001922 |
|  | scaRNA | 3 | 180 | 540 | 0.000049 |
|  | Unknown | 1 | 235 | 235 | 0.000021 |

**Supplementary Table 12.** Comparison of *S. oblata* genome

| Parameter | S. oblata (self) | S. oblata (Bo Ma et al., 2022) | S. oblata (Yi Wang et al., 2022) |
| --- | --- | --- | --- |
| Assembled genome size (Gb) | 1.11 | 1.12 | 1.12 |
| Chromosome-anchored scaffolds (Gb) | 1.10 | 1.05 | 1.12 |
| N50 of contigs (Mb) | 4.75 | 3.96 | 1.26 |
| Longest contigs (Mb) | 21.49 | 12.69 | 7.16 |
| N50 of scaffolds (Mp) | 44.71 | 46.33 | 49.73 |
| Longest scaffolds (Mb) | 70.97 | 69.21 | 77.6 |

**Supplementary Table 13.** Alignment rate of published *S. oblata* transcriptome to reference genome

| SRX/SRR ID. | *S. oblata* (self) | | | *S. oblata* (Yi Wang et al., 2022) | | |
| --- | --- | --- | --- | --- | --- | --- |
|  | Overall alignment rate | Aligned 1 time | Aligned >1 times | Overall alignment rate | Aligned 1 time | Aligned >1 times |
| SRX12379523 | 82.09% | 79.00% | 3.09% | 80.22% | 75.42% | 4.80% |
| SRX12379524 | 82.02% | 78.94% | 3.08% | 80.19% | 75.40% | 4.79% |
| SRX12379525 | 44.46% | 42.71% | 1.75% | 43.58% | 40.82% | 2.76% |
| SRX12379526 | 43.10% | 41.40% | 1.70% | 42.24% | 39.54% | 2.70% |
| SRX12379527 | 43.46% | 41.75% | 1.71% | 42.61% | 39.91% | 2.70% |
| SRX12379528 | 87.53% | 83.99% | 3.54% | 85.81% | 80.77% | 5.04% |
| SRX12379529 | 87.44% | 83.90% | 3.54% | 85.72% | 80.61% | 5.11% |
| SRX12379530 | 87.49% | 83.94% | 3.56% | 85.73% | 80.68% | 5.05% |
| SRX12379531 | 90.38% | 86.70% | 3.68% | 88.25% | 82.21% | 6.04% |
| SRX12379532 | 90.29% | 86.55% | 3.74% | 88.19% | 82.11% | 6.08% |
| SRX12379533 | 90.44% | 86.72% | 3.72% | 88.14% | 82.18% | 5.96% |
| SRR2474554 | 92.44% | 83.31% | 4.74% | 90.51% | 80.19% | 5.69% |
| SRR2474555 | 92.32% | 83.69% | 4.01% | 90.23% | 80.26% | 5.11% |
| SRR2474556 | 92.35% | 83.56% | 4.22% | 90.39% | 80.19% | 5.31% |
| SRR2475365 | 91.96% | 83.47% | 3.86% | 90.28% | 80.23% | 5.22% |
| SRR2475366 | 89.33% | 80.10% | 4.33% | 87.59% | 76.55% | 5.95% |
| SRR2475367 | 91.60% | 80.94% | 5.73% | 89.98% | 78.25% | 6.37% |
| SRR2475368 | 92.51% | 84.07% | 3.87% | 90.77% | 80.79% | 5.23% |
| SRR2475369 | 92.09% | 83.83% | 3.54% | 90.37% | 80.28% | 5.27% |
| SRR2475370 | 92.52% | 84.32% | 3.62% | 90.54% | 80.56% | 5.13% |

**Supplementary Table 14.** KEGG annotation of expansion genes

| MapID | MapTitle | Gene-number |
| --- | --- | --- |
| map03430 | Mismatch repair | 163 |
| map03420 | Nucleotide excision repair | 171 |
| map03030 | DNA replication | 164 |
| map03460 | Fanconi anemia pathway | 163 |
| map03440 | Homologous recombination | 163 |
| map05130 | Pathogenic Escherichia coli infection | 75 |
| map00460 | Cyanoamino acid metabolism | 63 |
| map00640 | Propanoate metabolism | 39 |
| map00720 | Carbon fixation pathways in prokaryotes | 41 |
| map00061 | Fatty acid biosynthesis | 40 |
| map00220 | Arginine biosynthesis | 31 |
| map00950 | Isoquinoline alkaloid biosynthesis | 35 |
| map01212 | Fatty acid metabolism | 40 |
| map03040 | Spliceosome | 81 |
| map00620 | Pyruvate metabolism | 46 |
| map00360 | Phenylalanine metabolism | 35 |
| map00940 | Phenylpropanoid biosynthesis | 63 |
| map01210 | 2-Oxocarboxylic acid metabolism | 31 |
| map00350 | Tyrosine metabolism | 27 |
| map05110 | Vibrio cholerae infection | 23 |
| map00960 | Tropane, piperidine and pyridine alkaloid biosynthesis | 21 |
| map00401 | Novobiocin biosynthesis | 14 |
| map00270 | Cysteine and methionine metabolism | 42 |
| map00130 | Ubiquinone and other terpenoid-quinone biosynthesis | 22 |
| map00909 | Sesquiterpenoid and triterpenoid biosynthesis | 17 |
| map01130 | Biosynthesis of antibiotics | 102 |
| map04392 | Hippo signaling pathway - multiple species | 11 |
| map00500 | Starch and sucrose metabolism | 55 |
| map00400 | Phenylalanine, tyrosine and tryptophan biosynthesis | 21 |
| map04975 | Fat digestion and absorption | 7 |
| map00901 | Indole alkaloid biosynthesis | 6 |
| map01110 | Biosynthesis of secondary metabolites | 213 |
| map05134 | Legionellosis | 25 |
| map01230 | Biosynthesis of amino acids | 58 |
| map04072 | Phospholipase D signaling pathway | 18 |
| map04138 | Autophagy - yeast | 27 |
| map01200 | Carbon metabolism | 54 |
| map00627 | Aminobenzoate degradation | 8 |
| map00523 | Polyketide sugar unit biosynthesis | 2 |
| map00643 | Styrene degradation | 8 |
| map05322 | Systemic lupus erythematosus | 14 |
| map00330 | Arginine and proline metabolism | 18 |
| map04726 | Serotonergic synapse | 7 |
| map00930 | Caprolactam degradation | 3 |
| map00965 | Betalain biosynthesis | 6 |
| map05030 | Cocaine addiction | 6 |
| map04966 | Collecting duct acid secretion | 7 |

**Supplementary Table 15.** KEGG annotation of *S. oblata*-specific genes

| MapID | MapTitle | Gene numbers |
| --- | --- | --- |
| map00909 | Sesquiterpenoid and triterpenoid biosynthesis | 28 |
| map00940 | Phenylpropanoid biosynthesis | 72 |
| map01220 | Degradation of aromatic compounds | 6 |
| map05130 | Pathogenic Escherichia coli infection | 71 |
| map03430 | Mismatch repair | 105 |
| map03030 | DNA replication | 107 |
| map03460 | Fanconi anemia pathway | 105 |
| map03440 | Homologous recombination | 105 |
| map03420 | Nucleotide excision repair | 105 |
| map00460 | Cyanoamino acid metabolism | 67 |
| map03040 | Spliceosome | 95 |
| map00130 | Ubiquinone and other terpenoid-quinone biosynthesis | 33 |
| map00220 | Arginine biosynthesis | 27 |
| map00401 | Novobiocin biosynthesis | 21 |
| map01210 | 2-Oxocarboxylic acid metabolism | 34 |
| map00960 | Tropane, piperidine and pyridine alkaloid biosynthesis | 24 |
| map00500 | Starch and sucrose metabolism | 69 |
| map00950 | Isoquinoline alkaloid biosynthesis | 28 |
| map00360 | Phenylalanine metabolism | 30 |
| map00400 | Phenylalanine, tyrosine and tryptophan biosynthesis | 26 |
| map00350 | Tyrosine metabolism | 26 |
| map01230 | Biosynthesis of amino acids | 68 |
| map00930 | Caprolactam degradation | 6 |
| map01524 | Platinum drug resistance | 22 |
| map00270 | Cysteine and methionine metabolism | 41 |
| map00361 | Chlorocyclohexane and chlorobenzene degradation | 4 |
| map00364 | Fluorobenzoate degradation | 4 |
| map00623 | Toluene degradation | 4 |
| map04350 | TGF-beta signaling pathway | 14 |
| map04979 | Cholesterol metabolism | 6 |
| map05016 | Huntington disease | 36 |
| map04260 | Cardiac muscle contraction | 8 |
| map04216 | Ferroptosis | 9 |
| map02020 | Two-component system | 6 |
| map00254 | Aflatoxin biosynthesis | 2 |
| map05012 | Parkinson disease | 22 |
| map01110 | Biosynthesis of secondary metabolites | 217 |
| map04012 | ErbB signaling pathway | 7 |
| map05206 | MicroRNAs in cancer | 20 |
| map05225 | Hepatocellular carcinoma | 17 |

**Supplementary Table 16. genes involved in the biosynthesis of cyanidin-3-O-rutinoside and delphinidin-3-O-rutinoside**

|  | *S. oblata* | *F. pennsylvanica* | *O. europaea* | *J. sambac* | *P. axillaris* | *P. inflata* |
| --- | --- | --- | --- | --- | --- | --- |
| gene-name | gene-id | gene-id | gene-id | gene-id | gene-id | gene-id |
| 4CL | evm.model.Chr1.3120 | Fp_g13978 | GWHPAOPM000369 | JS11279 | Peaxi162Scf00089g00045.1 | Peinf101Scf00536g08013.1 |
|  | evm.model.Chr1.4246 | Fp_g20592 | GWHPAOPM000532 | JS14934 | Peaxi162Scf00166g00529.1 | Peinf101Scf00663g00014.1 |
|  | evm.model.Chr10.234 | Fp_g21154 | GWHPAOPM000958 | JS1570 | Peaxi162Scf00195g01223.1 | Peinf101Scf00918g03006.1 |
|  | evm.model.Chr10.235 | Fp_g227 | GWHPAOPM011319 | JS17345 | Peaxi162Scf00207g00334.1 | Peinf101Scf01099g07012.1 |
|  | evm.model.Chr10.431 | Fp_g27253 | GWHPAOPM015809 | JS1748 | Peaxi162Scf00314g00086.1 | Peinf101Scf01230g00016.1 |
|  | evm.model.Chr16.1564 | Fp_g29654 | GWHPAOPM016834 | JS19775 | Peaxi162Scf00408g00018.1 | Peinf101Scf01482g05046.1 |
|  | evm.model.Chr18.1637 | Fp_g3820 | GWHPAOPM020205 | JS19948 | Peaxi162Scf00610g00346.1 | Peinf101Scf01633g10028.1 |
|  | evm.model.Chr19.371 | Fp_g38680 | GWHPAOPM021044 | JS20539 | Peaxi162Scf00682g00113.1 | Peinf101Scf01889g04030.1 |
|  | evm.model.Chr19.907 | Fp_g38827 | GWHPAOPM024561 | JS4233 | Peaxi162Scf00745g00865.1 | Peinf101Scf01969g01033.1 |
|  | evm.model.Chr2.2674 | Fp_g38828 | GWHPAOPM034550 | JS4799 |  | Peinf101Scf02008g02022.1 |
|  | evm.model.Chr20.1712 | Fp_g38829 | GWHPAOPM036025 | JS486 |  | Peinf101Scf04500g00008.1 |
|  | *evm.model.Chr4.68.2 | Fp_g42769 | GWHPAOPM036136 | JS487 |  |  |
|  | evm.model.Chr5.3187 | Fp_g6714 | GWHPAOPM037450 | JS488 |  |  |
|  | evm.model.Chr5.411 | Fp_g6715 | GWHPAOPM039364 | JS8559 |  |  |
|  | evm.model.Chr7.2828 | Fp_g8640 | GWHPAOPM048509 | |  |  |
|  | evm.model.Chr8.2012 |  |  |  |  |  |
|  | *evm.model.Chr9.394 |  |  |  |  |  |
| ANS | evm.model.Chr16.115 | Fp_g28144 | GWHPAOPM041094 | JS2292 | Peaxi162Scf00620g00533.1 | Peinf101Scf01166g06033.1 |
|  | *evm.model.Chr6.867 |  |  | JS8082 |  |  |
| BZ1 | evm.model.Chr11.2413 | Fp_g18500 | GWHPAOPM003656 | JS15185 | Peaxi162Scf00050g00423.1 | Peinf101Scf00086g03003.1 |
|  | evm.model.Chr7.527 | Fp_g38141 | GWHPAOPM023232 | JS15186 | Peaxi162Scf00163g00081.1 | Peinf101Scf00192g01001.1 |
|  |  |  |  |  |  | Peinf101Scf00777g01016.1 |
| C4H | evm.model.Chr13.2163 | Fp_g18834 | GWHPAOPM003369 | JS16709 | Peaxi162Scf00390g00225.1 | Peinf101Ctg13394552g00001.1 |
|  |  | Fp_g33759 | GWHPAOPM003996 | JS2166 | Peaxi162Scf00556g00035.1 | Peinf101Ctg13550828g00002.1 |
|  |  | Fp_g33760 | GWHPAOPM006367 | | Peaxi162Scf00954g00024.1 | Peinf101Scf00296g05002.1 |
|  |  | Fp_g41539 | GWHPAOPM045406 | |  | Peinf101Scf00951g08008.1 |
|  |  |  |  |  |  | Peinf101Scf01430g00027.1 |
|  |  |  |  |  |  | Peinf101Scf03806g00033.1 |
| CHI | evm.model.Chr10.714 | Fp_g45600 | GWHPAOPM000817 | JS19245 | Peaxi162Scf00006g00088.1 | Peinf101Scf00071g13014.1 |
|  | evm.model.Chr20.2153 | Fp_g46589 | GWHPAOPM009269 | JS5087 | Peaxi162Scf00038g01957.1 | Peinf101Scf02573g02020.1 |
|  | evm.model.Chr20.2236 | Fp_g5223 | GWHPAOPM011920 | JS8961 | Peaxi162Scf00167g01221.1 | Peinf101Scf03176g00016.1 |
|  |  |  | GWHPAOPM035096 | JS9039 | Peaxi162Scf00486g00315.1 | Peinf101Scf06002g00045.1 |
| CHS | evm.model.Chr14.573 | Fp_g1870 | GWHPAOPM006998 | JS13739 | Peaxi162Scf00047g01225.1 | Peinf101Scf00187g04018.1 |
|  | evm.model.Chr18.955 | Fp_g26142 | GWHPAOPM012414 | JS2277 | Peaxi162Scf00126g01326.1 | Peinf101Scf00437g05004.1 |
|  | evm.model.Chr19.1982 | Fp_g33173 | GWHPAOPM030733 | | Peaxi162Scf00164g00313.1 | Peinf101Scf00437g12010.1 |
|  | evm.model.Chr2.3288 | Fp_g34320 | GWHPAOPM050685 | | Peaxi162Scf00536g00092.1 | Peinf101Scf00559g12002.1 |
|  | evm.model.Chr4.3107 |  | GWHPAOPM051864 | | Peaxi162Scf00817g00216.1 | Peinf101Scf00631g01013.1 |
|  | evm.model.Chr9.2339 |  |  |  | Peaxi162Scf00830g00016.1 | Peinf101Scf01775g10041.1 |
|  |  |  |  |  | Peaxi162Scf00830g00048.1 | Peinf101Scf01775g10050.1 |
|  |  |  |  |  | Peaxi162Scf00830g00067.1 | |
|  |  |  |  |  | Peaxi162Scf00938g00001.1 | |
|  |  |  |  |  | Peaxi162Scf00938g00118.1 | |
|  |  |  |  |  | Peaxi162Scf01698g00005.1 | |
|  |  |  |  |  | Peaxi162Scf08277g00001.1 | |
| DFR | evm.model.Chr11.847 | Fp_g19769 | GWHPAOPM002769 | JS17607 | Peaxi162Scf00366g00630.1 | Peinf101Scf00073g04027.1 |
|  | evm.model.Chr15.651 |  | GWHPAOPM013111 | JS20031 |  | Peinf101Scf00590g21022.1 |
|  | evm.model.Chr20.1657 |  |  | JS8539 |  |  |
|  | evm.model.Chr8.1144 |  |  |  |  |  |
| F3'5'H | *evm.model.Chr6.845_evm.model.Chr6.846 | Fp_g28128 | GWHPAOPM041111 | JS8052 | Peinf101Scf00586g11035.1 | Peinf101Scf00872g01009.1 |
| F3H | evm.model.Chr1.3090 | Fp_g1136 | GWHPAOPM013847 | JS17406 | Peaxi162Scf00328g01214.1 | Peinf101Scf01063g07016.1 |
|  | evm.model.Chr12.2312 | Fp_g15734 | GWHPAOPM013885 | |  |  |
|  | evm.model.Chr12.621 | Fp_g15735 | GWHPAOPM050579 | |  |  |
|  | *evm.model.Chr13.484 | Fp_g15736 | GWHPAOPM052721 | |  |  |
|  | evm.model.Chr13.485 | Fp_g15737 |  |  |  |  |
|  | *evm.model.Chr13.560 |  |  |  |  |  |
|  | evm.model.Chr9.464 |  |  |  |  |  |
| F3'H | evm.model.Chr1.2169 | Fp_g1827 | GWHPAOPM018138 | JS9612 | Peaxi162Scf00201g00243.1 | Peinf101Scf01556g04023.1 |
|  | *evm.model.Chr7.2414 |  |  |  |  |  |
| PAL | evm.model.Chr1.1010 | Fp_g11352 | GWHPAOPM006627 | JS6395 | Peaxi162Scf00123g00096.1 | Peinf101Scf00622g01006.1 |
|  | *evm.model.Chr16.1894 | Fp_g11354 | GWHPAOPM006631 | JS6396 | Peaxi162Scf00488g00074.1 | Peinf101Scf00871g09016.1 |
|  | *evm.model.Chr16.1895 | Fp_g13359 | GWHPAOPM017116 | JS6397 | Peaxi162Scf00858g00215.1 | Peinf101Scf01985g02016.1 |
|  | evm.model.Chr4.3315 | Fp_g19252 | GWHPAOPM021770 | JS777 |  |  |
|  | evm.model.Chr4.759 | Fp_g19253 | GWHPAOPM023790 | |  |  |
|  |  | Fp_g19254 | GWHPAOPM023796 | |  |  |
|  |  | Fp_g2819 | GWHPAOPM023802 | |  |  |
|  |  | Fp_g43086 | GWHPAOPM030795 | |  |  |
|  |  | Fp_g45355 | GWHPAOPM031349 | |  |  |
|  |  | Fp_g45356 | GWHPAOPM045048 | |  |  |
|  |  |  | GWHPAOPM049066 | |  |  |
|  |  |  | GWHPAOPM051211 | |  |  |
|  |  |  | GWHPAOPM053079 | |  |  |

* : Candidate genes affecting flower color differences among individuals of *S. oblata.*

**Supplementary Table 17. TPS gene family in *S. oblata* and relative species**

| TPS Subfamily | *S. oblata* | *A. thaliana* | *F. pennsylvanica* | *O. europaea* | *J. sambac* |
| --- | --- | --- | --- | --- | --- |
|  | Gene-id | Gene-id | Gene-id | Gene-id | Gene-id |
| TPS-a | evm.model.Chr1.544 | AT1G31950.1 | Fp_g20132 | GWHPAOPM009669 | JS11797 |
|  | evm.model.Chr1.587 | AT1G33750.1 | Fp_g3265 | GWHPAOPM009691 | JS11798 |
|  | evm.model.Chr10.2004 | AT1G48800.1 | Fp_g3268 | GWHPAOPM009695 | JS12287 |
|  | evm.model.Chr2.294 | AT1G48820.1 | Fp_g3290 | GWHPAOPM009696 | JS14294 |
|  | evm.model.Chr2.524.4 | AT1G66020.1 | Fp_g38179 | GWHPAOPM009697 | JS14296 |
|  | evm.model.Chr2.532 | AT1G70080.1 | Fp_g44498 | GWHPAOPM009722 | JS15218 |
|  | evm.model.Chr20.675 | AT2G23230.1 | Fp_g44499 | GWHPAOPM009723 | JS18987 |
|  | evm.model.Chr21.522 | AT2G37140.1 | Fp_g46756 | GWHPAOPM009918 | JS4158 |
|  | evm.model.Chr23.442 | AT3G14490.1 | Fp_g49438 | GWHPAOPM009925 | JS4897 |
|  | evm.model.Chr23.451 | AT3G14520.1 | Fp_g49440 | GWHPAOPM009928 | JS5839 |
|  | evm.model.Chr23.559 | AT3G14540.1 | Fp_g49441 | GWHPAOPM009931 | JS5840 |
|  |  | AT3G29110.1 | Fp_g49463 | GWHPAOPM019475 | JS5843 |
|  |  | AT3G29190.1 | Fp_g6132 | GWHPAOPM019492 | JS5844 |
|  |  | AT3G29410.1 | Fp_g6133 | GWHPAOPM020718 | JS8485 |
|  |  | AT3G32030.1 | Fp_g6137 | GWHPAOPM020719 | JS8492 |
|  |  | AT4G13280.1 | Fp_g6167 | GWHPAOPM020722 | JS8493 |
|  |  | AT4G13300.1 | Fp_g6169 | GWHPAOPM020730 | JS8496 |
|  |  | AT4G15870.1 | Fp_g6171 | GWHPAOPM020731 | JS913 |
|  |  | AT4G20200.1 | Fp_g6400 | GWHPAOPM044756 | JS914 |
|  |  | AT4G20210.1 | Fp_g760 | GWHPAOPM047361 | JS921 |
|  |  | AT4G20230.1 |  | GWHPAOPM047362 | JS922 |
|  |  | AT5G23960.1 |  |  | JS927 |
|  |  | AT5G23960.2 |  |  | JS928 |
|  |  | AT5G44630.1 |  |  |  |
|  |  | AT5G48110.1 |  |  |  |
| TPS-b | evm.model.Chr12.2715 | AT2G24210.1 | Fp_g10397 | GWHPAOPM010308 | JS15626 |
|  | evm.model.Chr12.2718 | AT3G25810.1 | Fp_g10398 | GWHPAOPM027479 | JS15628 |
|  | evm.model.Chr12.2731 | AT3G25820.1 | Fp_g10401 | GWHPAOPM027486 | JS15631 |
|  | evm.model.Chr12.2765 | AT3G25820.2 | Fp_g22825 | GWHPAOPM027488 | JS5554 |
|  | evm.model.Chr15.2295 | AT3G25830.1 | Fp_g24865 | GWHPAOPM027490 | JS5562 |
|  | evm.model.Chr21.1733 | AT4G16730.1 | Fp_g44126 | GWHPAOPM027498 | JS5564 |
|  | evm.model.Chr21.1752 | AT4G16740.1 | Fp_g5607 | GWHPAOPM027501 | JS7610 |
|  | evm.model.Chr23.944 | AT4G16740.2 | Fp_g9145 | GWHPAOPM027513 | JS7615 |
|  | evm.model.Chr3.2522 | |  | GWHPAOPM027516 |  |
|  | evm.model.Chr3.2523 | |  | GWHPAOPM027517 |  |
|  | evm.model.Chr3.2524 | |  | GWHPAOPM031982 |  |
|  | evm.model.Chr3.698 | |  | GWHPAOPM031983 |  |
|  |  |  |  | GWHPAOPM031986 |  |
|  |  |  |  | GWHPAOPM031987 |  |
|  |  |  |  | GWHPAOPM031988 |  |
|  |  |  |  | GWHPAOPM031989 |  |
|  |  |  |  | GWHPAOPM031990 |  |
|  |  |  |  | GWHPAOPM032730 |  |
|  |  |  |  | GWHPAOPM032732 |  |
|  |  |  |  | GWHPAOPM032734 |  |
|  |  |  |  | GWHPAOPM032735 |  |
|  |  |  |  | GWHPAOPM032736 |  |
|  |  |  |  | GWHPAOPM032737 |  |
|  |  |  |  | GWHPAOPM044407 |  |
| TPS-c | evm.model.Chr14.677 | AT4G02780.1 | Fp_g32443 | GWHPAOPM007727 | JS2946 |
|  | evm.model.Chr2.3108 | | Fp_g40256 | GWHPAOPM007733 | JS4887 |
|  | evm.model.Chr20.2151 | | Fp_g4195 | GWHPAOPM007734 | JS8404 |
|  | evm.model.Chr22.1652 | | Fp_g45312 | GWHPAOPM007735 | JS8948 |
|  |  |  |  | GWHPAOPM009206 |  |
|  |  |  |  | GWHPAOPM011718 |  |
|  |  |  |  | GWHPAOPM014759 |  |
|  |  |  |  | GWHPAOPM035016 |  |
|  |  |  |  | GWHPAOPM043240 |  |
| TPS-e | evm.model.Chr14.678 | AT1G79460.1 | Fp_g32446 | GWHPAOPM044583 | JS12289 |
|  | evm.model.Chr23.669 | | Fp_g40134 |  | JS2926 |
| TPS-f | evm.model.Chr2.1417 | AT1G61120.1 | Fp_g44068 | GWHPAOPM009936 | JS12118 |
|  | evm.model.Chr23.1044 | | Fp_g5501 | GWHPAOPM010399 |  |
| TPS-g | evm.model.Chr1.155 | AT1G61680.1 | Fp_g28626 | GWHPAOPM010318 | JS10756 |
|  | evm.model.Chr2.1313 | AT1G61680.2 | Fp_g41054 | GWHPAOPM010320 | JS10760 |
|  | evm.model.Chr2.1318 | | Fp_g41745 | GWHPAOPM010323 | JS11395 |
|  | evm.model.Chr20.1271 | | Fp_g43770 | GWHPAOPM010324 | JS11938 |
|  | evm.model.Chr23.1669 | | Fp_g43772 | GWHPAOPM010326 | JS5553 |
|  | evm.model.Chr6.1332 | | Fp_g5571 | GWHPAOPM010327 | JS7413 |
|  |  |  | Fp_g5576 | GWHPAOPM010328 | JS7415 |
|  |  |  | Fp_g5588 | GWHPAOPM010329 |  |
|  |  |  | Fp_g5589 | GWHPAOPM010330 |  |
|  |  |  | Fp_g5590 | GWHPAOPM026977 |  |
|  |  |  |  | GWHPAOPM033914 |  |
|  |  |  |  | GWHPAOPM033915 |  |
|  |  |  |  | GWHPAOPM033927 |  |
|  |  |  |  | GWHPAOPM040584 |  |
|  |  |  |  | GWHPAOPM040585 |  |
|  |  |  |  | GWHPAOPM043847 |  |
|  |  |  |  | GWHPAOPM043848 |  |
|  |  |  |  | GWHPAOPM050983 |  |

**Supplementary Table 18. CYP450 genes in *S. oblata* and and *A. thaliana***

| *A. thaliana* | | | | *S. oblata* | | | |
| --- | --- | --- | --- | --- | --- | --- | --- |
| Clan | Family | Subfamily | gene-id | Clan | Family | Subfamily | gene-id |
| Clan51 | CYP51 | CYP51A | AT1G11680.1 | Clan51 | CYP51 | CYP51A | evm.model.Chr10.2937 |
| Clan71 | CYP701 | CYP701A | AT5G25900.1 | Clan51 | CYP51 | CYP51A | evm.model.Chr5.342 |
| Clan71 | CYP703 | CYP703A2 | AT1G01280.1 | Clan71 | CYP701 | CYP701A | evm.model.Chr15.1704 |
| Clan71 | CYP705 | CYP705A | AT1G28430.1 | Clan71 | CYP701 | CYP701A | evm.model.Chr9.643 |
| Clan71 | CYP705 | CYP705A | AT1G50520.1 | Clan71 | CYP703 |  | evm.model.Chr20.1684 |
| Clan71 | CYP705 | CYP705A | AT1G50560.1 | Clan71 | CYP706 | CYP706A | evm.model.Chr14.123 |
| Clan71 | CYP705 | CYP705A | AT2G05180.1 | Clan71 | CYP706 | CYP706A | evm.model.Chr6.845 |
| Clan71 | CYP705 | CYP705A | AT2G14100.1 | Clan71 | CYP71 | CYP71A | evm.model.Chr1.1874 |
| Clan71 | CYP705 | CYP705A | AT2G27000.1 | Clan71 | CYP71 | CYP71A | evm.model.Chr1.874 |
| Clan71 | CYP705 | CYP705A | AT2G27010.1 | Clan71 | CYP71 | CYP71A | evm.model.Chr10.408 |
| Clan71 | CYP705 | CYP705A | AT3G20080.1 | Clan71 | CYP71 | CYP71A | evm.model.Chr13.1873 |
| Clan71 | CYP705 | CYP705A | AT3G20090.2 | Clan71 | CYP71 | CYP71A | evm.model.Chr5.2988 |
| Clan71 | CYP705 | CYP705A | AT3G20100.1 | Clan71 | CYP71 | CYP71A | evm.model.Chr5.2990 |
| Clan71 | CYP705 | CYP705A | AT3G20110.1 | Clan71 | CYP71 | CYP71A | evm.model.Chr5.2991 |
| Clan71 | CYP705 | CYP705A | AT3G20120.3 | Clan71 | CYP71 | CYP71A | evm.model.Chr6.2713 |
| Clan71 | CYP705 | CYP705A | AT3G20130.1 | Clan71 | CYP71 | CYP71A | evm.model.Chr7.1170 |
| Clan71 | CYP705 | CYP705A | AT3G20140.1 | Clan71 | CYP71 | CYP71A | evm.model.Chr7.1176 |
| Clan71 | CYP705 | CYP705A | AT3G20935.1 | Clan71 | CYP71 | CYP71A | evm.model.Chr7.2744 |
| Clan71 | CYP705 | CYP705A | AT3G20940.1 | Clan71 | CYP71 | CYP71A | evm.model.Chr7.2757 |
| Clan71 | CYP705 | CYP705A | AT3G20950.1 | Clan71 | CYP71 | CYP71A | evm.model.Chr7.2758 |
| Clan71 | CYP705 | CYP705A | AT3G20960.2 | Clan71 | CYP71 | CYP71A | evm.model.Chr7.2771 |
| Clan71 | CYP705 | CYP705A | AT4G15330.1 | Clan71 | CYP71 | CYP71B | evm.model.Chr1.1872 |
| Clan71 | CYP705 | CYP705A | AT4G15350.1 | Clan71 | CYP71 | CYP71B | evm.model.Chr1.1873 |
| Clan71 | CYP705 | CYP705A | AT4G15360.1 | Clan71 | CYP71 | CYP71B | evm.model.Chr1.3586 |
| Clan71 | CYP705 | CYP705A | AT4G15380.1 | Clan71 | CYP71 | CYP71B | evm.model.Chr13.2161 |
| Clan71 | CYP705 | CYP705A | AT5G42580.1 | Clan71 | CYP71 | CYP71D | evm.model.Chr13.269 |
| Clan71 | CYP705 | CYP705A | AT5G47990.1 | Clan71 | CYP71 | CYP71D | evm.model.Chr13.291 |
| Clan71 | CYP706 | CYP706A | AT4G12300.1 | Clan71 | CYP71 | CYP71D | evm.model.Chr13.295 |
| Clan71 | CYP706 | CYP706A | AT4G12310.1 | Clan71 | CYP71 | CYP71D | evm.model.Chr15.1148 |
| Clan71 | CYP706 | CYP706A | AT4G12320.1 | Clan71 | CYP71 | CYP71D | evm.model.Chr2.1056 |
| Clan71 | CYP706 | CYP706A | AT4G12330.1 | Clan71 | CYP71 | CYP71D | evm.model.Chr2.3101 |
| Clan71 | CYP706 | CYP706A | AT4G22690.1 | Clan71 | CYP71 | CYP71D | evm.model.Chr2.3107 |
| Clan71 | CYP706 | CYP706A | AT4G22710.1 | Clan71 | CYP71 | CYP71D | evm.model.Chr21.1721 |
| Clan71 | CYP706 | CYP706A | AT5G44620.1 | Clan71 | CYP71 | CYP71D | evm.model.Chr21.1738 |
| Clan71 | CYP71 | CYP71A | AT1G11610.2 | Clan71 | CYP71 | CYP71D | evm.model.Chr21.1761 |
| Clan71 | CYP71 | CYP71A | AT2G30750.1 | Clan71 | CYP71 | CYP71D | evm.model.Chr21.1768 |
| Clan71 | CYP71 | CYP71A | AT2G30770.1 | Clan71 | CYP71 | CYP71D | evm.model.Chr23.758 |
| Clan71 | CYP71 | CYP71A | AT3G48270.1 | Clan71 | CYP71 | CYP71D | evm.model.Chr23.759 |
| Clan71 | CYP71 | CYP71A | AT3G48280.1 | Clan71 | CYP71 | CYP71D | evm.model.Chr3.1711 |
| Clan71 | CYP71 | CYP71A | AT3G48290.2 | Clan71 | CYP71 | CYP71D | evm.model.Chr3.1906 |
| Clan71 | CYP71 | CYP71A | AT3G48300.2 | Clan71 | CYP71 | CYP71D | evm.model.Chr6.661 |
| Clan71 | CYP71 | CYP71A | AT3G48310.1 | Clan71 | CYP71 | CYP71D | evm.model.Chr6.663 |
| Clan71 | CYP71 | CYP71A | AT3G48320.1 | Clan71 | CYP71 | CYP71D | evm.model.Chr6.675 |
| Clan71 | CYP71 | CYP71A | AT4G13290.1 | Clan71 | CYP71 | CYP71D | evm.model.Chr6.692 |
| Clan71 | CYP71 | CYP71A | AT4G13310.1 | Clan71 | CYP71 | CYP71D | evm.model.Chr6.694 |
| Clan71 | CYP71 | CYP71A | AT4G20235.1 | Clan71 | CYP712 | CYP712A | evm.model.Chr8.2843 |
| Clan71 | CYP71 | CYP71A | AT4G20240.1 | Clan71 | CYP73 | CYP73A | evm.model.Chr11.2078 |
| Clan71 | CYP71 | CYP71A | AT5G24950.1 | Clan71 | CYP73 | CYP73A | evm.model.Chr13.2163 |
| Clan71 | CYP71 | CYP71A | AT5G24960.1 | Clan71 | CYP75 | CYP75B | evm.model.Chr1.1355 |
| Clan71 | CYP71 | CYP71A | AT5G42590.1 | Clan71 | CYP75 | CYP75B | evm.model.Chr1.1358 |
| Clan71 | CYP71 | CYP71B | AT1G13080.1 | Clan71 | CYP75 | CYP75B | evm.model.Chr1.1360 |
| Clan71 | CYP71 | CYP71B | AT1G13090.2 | Clan71 | CYP75 | CYP75B | evm.model.Chr1.1361 |
| Clan71 | CYP71 | CYP71B | AT1G13100.1 | Clan71 | CYP75 | CYP75B | evm.model.Chr1.2169 |
| Clan71 | CYP71 | CYP71B | AT1G13110.1 | Clan71 | CYP75 | CYP75B | evm.model.Chr1.2440 |
| Clan71 | CYP71 | CYP71B | AT2G02580.1 | Clan71 | CYP75 | CYP75B | evm.model.Chr1.3650 |
| Clan71 | CYP71 | CYP71B | AT2G24180.1 | Clan71 | CYP75 | CYP75B | evm.model.Chr1.3656 |
| Clan71 | CYP71 | CYP71B | AT3G26150.1 | Clan71 | CYP75 | CYP75B | evm.model.Chr11.289 |
| Clan71 | CYP71 | CYP71B | AT3G26160.1 | Clan71 | CYP75 | CYP75B | evm.model.Chr11.290 |
| Clan71 | CYP71 | CYP71B | AT3G26170.1 | Clan71 | CYP75 | CYP75B | evm.model.Chr11.291 |
| Clan71 | CYP71 | CYP71B | AT3G26180.2 | Clan71 | CYP75 | CYP75B | evm.model.Chr12.811 |
| Clan71 | CYP71 | CYP71B | AT3G26190.1 | Clan71 | CYP75 | CYP75B | evm.model.Chr3.608 |
| Clan71 | CYP71 | CYP71B | AT3G26200.1 | Clan71 | CYP75 | CYP75B | evm.model.Chr7.2414 |
| Clan71 | CYP71 | CYP71B | AT3G26210.1 | Clan71 | CYP76 | CYP76A | evm.model.Chr10.299 |
| Clan71 | CYP71 | CYP71B | AT3G26220.1 | Clan71 | CYP76 | CYP76A | evm.model.Chr3.411 |
| Clan71 | CYP71 | CYP71B | AT3G26230.1 | Clan71 | CYP76 | CYP76A | evm.model.Chr4.3419 |
| Clan71 | CYP71 | CYP71B | AT3G26270.1 | Clan71 | CYP76 | CYP76A | evm.model.Chr4.3421 |
| Clan71 | CYP71 | CYP71B | AT3G26280.1 | Clan71 | CYP76 | CYP76A | evm.model.Chr8.1813 |
| Clan71 | CYP71 | CYP71B | AT3G26290.2 | Clan71 | CYP76 | CYP76A | evm.model.Chr9.2464 |
| Clan71 | CYP71 | CYP71B | AT3G26300.1 | Clan71 | CYP76 | CYP76C | evm.model.Chr10.297 |
| Clan71 | CYP71 | CYP71B | AT3G26310.1 | Clan71 | CYP76 | CYP76C | evm.model.Chr10.305 |
| Clan71 | CYP71 | CYP71B | AT3G26320.1 | Clan71 | CYP76 | CYP76C | evm.model.Chr12.2394 |
| Clan71 | CYP71 | CYP71B | AT3G26330.1 | Clan71 | CYP76 | CYP76C | evm.model.Chr12.2395 |
| Clan71 | CYP71 | CYP71B | AT3G26830.1 | Clan71 | CYP76 | CYP76C | evm.model.Chr12.2396 |
| Clan71 | CYP71 | CYP71B | AT3G44250.1 | Clan71 | CYP76 | CYP76C | evm.model.Chr17.680 |
| Clan71 | CYP71 | CYP71B | AT3G53280.1 | Clan71 | CYP76 | CYP76C | evm.model.Chr9.721 |
| Clan71 | CYP71 | CYP71B | AT3G53290.1 | Clan71 | CYP76 | CYP76G | evm.model.Chr13.136 |
| Clan71 | CYP71 | CYP71B | AT3G53300.1 | Clan71 | CYP77 | CYP77A | evm.model.Chr10.614 |
| Clan71 | CYP71 | CYP71B | AT3G53305.1 | Clan71 | CYP77 | CYP77A | evm.model.Chr17.1294 |
| Clan71 | CYP71 | CYP71B | AT5G25120.1 | Clan71 | CYP77 | CYP77A | evm.model.Chr5.2760 |
| Clan71 | CYP71 | CYP71B | AT5G25130.1 | Clan71 | CYP77 | CYP77B | evm.model.Chr2.1272 |
| Clan71 | CYP71 | CYP71B | AT5G25140.1 | Clan71 | CYP77 | CYP77B | evm.model.Chr23.896 |
| Clan71 | CYP71 | CYP71B | AT5G25180.1 | Clan71 | CYP78 | CYP78A | evm.model.Chr10.2884 |
| Clan71 | CYP71 | CYP71B | AT5G35715.1 | Clan71 | CYP78 | CYP78A | evm.model.Chr11.236 |
| Clan71 | CYP71 | CYP71B | AT5G57260.1 | Clan71 | CYP78 | CYP78A | evm.model.Chr12.1433 |
| Clan71 | CYP712 | CYP712A | AT2G42250.1 | Clan71 | CYP78 | CYP78A | evm.model.Chr12.63 |
| Clan71 | CYP712 | CYP712A | AT5G06905.1 | Clan71 | CYP78 | CYP78A | evm.model.Chr15.609 |
| Clan71 | CYP73 | CYP73A | AT2G30490.1 | Clan71 | CYP78 | CYP78A | evm.model.Chr19.286 |
| Clan71 | CYP75 | CYP75B | AT5G07990.1 | Clan71 | CYP78 | CYP78A | evm.model.Chr20.1693 |
| Clan71 | CYP76 | CYP76C | AT1G33720.5 | Clan71 | CYP78 | CYP78A | evm.model.Chr21.2152 |
| Clan71 | CYP76 | CYP76C | AT1G33730.1 | Clan71 | CYP78 | CYP78A | evm.model.Chr5.236 |
| Clan71 | CYP76 | CYP76C | AT2G45550.1 | Clan71 | CYP78 | CYP78A | evm.model.Chr8.320 |
| Clan71 | CYP76 | CYP76C | AT2G45560.3 | Clan71 | CYP79 | CYP79A | evm.model.Chr5.2626 |
| Clan71 | CYP76 | CYP76C | AT2G45570.1 | Clan71 | CYP79 | CYP79B | evm.model.Chr11.1433 |
| Clan71 | CYP76 | CYP76C | AT2G45580.1 | Clan71 | CYP80 | CYP80G2 | evm.model.Chr2.2876 |
| Clan71 | CYP76 | CYP76C | AT3G61040.2 | Clan71 | CYP80 | CYP80G2 | evm.model.Chr2.3120 |
| Clan71 | CYP76 | CYP76G | AT3G52970.1 | Clan71 | CYP80 | CYP80G2 | evm.model.Chr6.2664 |
| Clan71 | CYP77 | CYP77A | AT3G10560.1 | Clan71 | CYP80 | CYP80G2 | evm.model.Chr6.2687 |
| Clan71 | CYP77 | CYP77A | AT3G10570.1 | Clan71 | CYP80 | CYP80G2 | evm.model.Chr6.2696 |
| Clan71 | CYP77 | CYP77A | AT3G18270.1 | Clan71 | CYP81 | CYP81D | evm.model.Chr2.3396 |
| Clan71 | CYP77 | CYP77A | AT5G04630.1 | Clan71 | CYP81 | CYP81D | evm.model.Chr20.2060 |
| Clan71 | CYP77 | CYP77A | AT5G04660.1 | Clan71 | CYP81 | CYP81E | evm.model.Chr1.3997 |
| Clan71 | CYP77 | CYP77B | AT1G11600.1 | Clan71 | CYP81 | CYP81E | evm.model.Chr13.922 |
| Clan71 | CYP78 | CYP78A | AT1G01190.1 | Clan71 | CYP81 | CYP81E | evm.model.Chr6.2844 |
| Clan71 | CYP78 | CYP78A | AT1G13710.1 | Clan71 | CYP81 | CYP81E | evm.model.Chr9.1790 |
| Clan71 | CYP78 | CYP78A | AT1G74110.1 | Clan71 | CYP81 | CYP81F | evm.model.Chr13.1022 |
| Clan71 | CYP78 | CYP78A | AT2G46660.1 | Clan71 | CYP81 | CYP81F | evm.model.Chr13.928 |
| Clan71 | CYP78 | CYP78A | AT3G61880.1 | Clan71 | CYP81 | CYP81F | evm.model.Chr13.929 |
| Clan71 | CYP78 | CYP78A | AT5G09970.1 | Clan71 | CYP81 | CYP81F | evm.model.Chr18.1647 |
| Clan71 | CYP79 | CYP79A | AT5G05260.2 | Clan71 | CYP81 | CYP81G | evm.model.Chr19.1534 |
| Clan71 | CYP79 | CYP79A | AT5G35917.1 | Clan71 | CYP81 | CYP81K | evm.model.Chr19.1529 |
| Clan71 | CYP79 | CYP79A | AT5G35920.1 | Clan71 | CYP81 | CYP81K | evm.model.Chr9.1798 |
| Clan71 | CYP79 | CYP79B | AT2G22330.1 | Clan71 | CYP82 | CYP82C | evm.model.Chr13.2263 |
| Clan71 | CYP79 | CYP79B | AT4G39950.2 | Clan71 | CYP82 | CYP82C | evm.model.Chr22.1468 |
| Clan71 | CYP79 | CYP79C | AT1G58260.1 | Clan71 | CYP82 | CYP82C | evm.model.Chr5.2224 |
| Clan71 | CYP79 | CYP79C | AT1G79370.1 | Clan71 | CYP82 | CYP82C | evm.model.Chr9.635 |
| Clan71 | CYP79 | CYP79F | AT1G16400.1 | Clan71 | CYP82 | CYP82D | evm.model.Chr1.2994 |
| Clan71 | CYP79 | CYP79F | AT1G16410.1 | Clan71 | CYP82 | CYP82D | evm.model.Chr7.1497 |
| Clan71 | CYP81 | CYP81D | AT2G23190.1 | Clan71 | CYP83 | CYP83A | evm.model.Chr14.1470 |
| Clan71 | CYP81 | CYP81D | AT2G23220.1 | Clan71 | CYP83 | CYP83B | evm.model.Chr11.1412 |
| Clan71 | CYP81 | CYP81D | AT3G28740.1 | Clan71 | CYP83 | CYP83B | evm.model.Chr3.217 |
| Clan71 | CYP81 | CYP81D | AT4G37320.1 | Clan71 | CYP83 | CYP83B | evm.model.Chr8.1693 |
| Clan71 | CYP81 | CYP81D | AT4G37330.1 | Clan71 | CYP84 | CYP84A | evm.model.Chr1.1475 |
| Clan71 | CYP81 | CYP81D | AT4G37340.1 | Clan71 | CYP84 | CYP84A | evm.model.Chr7.1797 |
| Clan71 | CYP81 | CYP81D | AT4G37360.1 | Clan71 | CYP89 | CYP89A | evm.model.Chr11.1029 |
| Clan71 | CYP81 | CYP81D | AT4G37370.1 | Clan71 | CYP89 | CYP89A | evm.model.Chr8.1269 |
| Clan71 | CYP81 | CYP81D1 | AT5G36220.1 | Clan71 | CYP93 | CYP93D | evm.model.Chr1.624 |
| Clan71 | CYP81 | CYP81F | AT4G37400.1 | Clan71 | CYP93 | CYP93D | evm.model.Chr4.216 |
| Clan71 | CYP81 | CYP81F | AT4G37410.1 | Clan71 | CYP98 | CYP98A | evm.model.Chr12.2182_evm.model.Chr12.2180 |
| Clan71 | CYP81 | CYP81F | AT4G37430.1 | Clan71 | CYP98 | CYP98A | evm.model.Chr2.664 |
| Clan71 | CYP81 | CYP81F | AT5G57220.1 | Clan71 | CYP98 | CYP98A | evm.model.Chr21.679 |
| Clan71 | CYP81 | CYP81G | AT5G67310.1 | Clan710 | CYP710 | CYP710A | evm.model.Chr20.506 |
| Clan71 | CYP81 | CYP81H | AT4G37310.1 | Clan710 | CYP710 | CYP710A | evm.model.Chr3.2727 |
| Clan71 | CYP81 | CYP81K | AT5G10600.1 | Clan710 | CYP710 | CYP710A | evm.model.Chr3.2747 |
| Clan71 | CYP81 | CYP81K | AT5G10610.1 | Clan711 | CYP711 | CYP711A | evm.model.Chr2.3283 |
| Clan71 | CYP82 | CYP82C | AT4G31940.1 | Clan72 | CYP714 | CYP714A | evm.model.Chr18.334 |
| Clan71 | CYP82 | CYP82C | AT4G31950.1 | Clan72 | CYP714 | CYP714A | evm.model.Chr20.1465 |
| Clan71 | CYP82 | CYP82C | AT4G31970.1 | Clan72 | CYP714 | CYP714A | evm.model.Chr22.1426 |
| Clan71 | CYP82 | CYP82F | AT2G25160.1 | Clan72 | CYP714 | CYP714A | evm.model.Chr4.2182 |
| Clan71 | CYP82 | CYP82G | AT3G25180.2 | Clan72 | CYP714 | CYP714A | evm.model.Chr4.376 |
| Clan71 | CYP83 | CYP83A | AT4G13770.1 | Clan72 | CYP714 | CYP714A | evm.model.Chr6.2421 |
| Clan71 | CYP83 | CYP83B | AT4G31500.1 | Clan72 | CYP714 | CYP714C | evm.model.Chr20.1464 |
| Clan71 | CYP84 | CYP84A | AT4G36220.1 | Clan72 | CYP715 | CYP715A | evm.model.Chr4.244 |
| Clan71 | CYP84 | CYP84A | AT5G04330.1 | Clan72 | CYP715 | CYP715A | evm.model.Chr5.2517 |
| Clan71 | CYP89 | CYP89A | AT1G64900.1 | Clan72 | CYP72 | CYP72A | evm.model.Chr1.927 |
| Clan71 | CYP89 | CYP89A | AT1G64930.1 | Clan72 | CYP72 | CYP72A | evm.model.Chr11.2021 |
| Clan71 | CYP89 | CYP89A | AT1G64940.1 | Clan72 | CYP72 | CYP72A | evm.model.Chr14.1416 |
| Clan71 | CYP89 | CYP89A | AT1G64950.1 | Clan72 | CYP72 | CYP72A | evm.model.Chr16.882 |
| Clan71 | CYP89 | CYP89A | AT3G03470.1 | Clan72 | CYP72 | CYP72A | evm.model.Chr2.188 |
| Clan71 | CYP89 | CYP89A | AT5G61320.1 | Clan72 | CYP72 | CYP72A | evm.model.Chr2.189 |
| Clan71 | CYP93 | CYP93D | AT5G06900.1 | Clan72 | CYP72 | CYP72A | evm.model.Chr2.2159 |
| Clan71 | CYP98 | CYP98A | AT1G74540.1 | Clan72 | CYP72 | CYP72A | evm.model.Chr2.252 |
| Clan71 | CYP98 | CYP98A | AT1G74550.1 | Clan72 | CYP72 | CYP72A | evm.model.Chr2.275 |
| Clan71 | CYP98 | CYP98A | AT2G40890.1 | Clan72 | CYP72 | CYP72A | evm.model.Chr23.172 |
| Clan710 | CYP710 | CYP710A | AT2G28850.1 | Clan72 | CYP72 | CYP72A | evm.model.Chr23.175 |
| Clan710 | CYP710 | CYP710A | AT2G28860.1 | Clan72 | CYP72 | CYP72A | evm.model.Chr23.274 |
| Clan710 | CYP710 | CYP710A | AT2G34490.1 | Clan72 | CYP72 | CYP72C | evm.model.Chr19.813 |
| Clan710 | CYP710 | CYP710A | AT2G34500.1 | Clan72 | CYP72 | CYP72C | evm.model.Chr4.3427 |
| Clan711 | CYP711 | CYP711A | AT2G26170.2 | Clan72 | CYP721 | CYP721A | evm.model.Chr1.3027 |
| Clan72 | CYP709 | CYP709B | AT2G46950.1 | Clan72 | CYP721 | CYP721A | evm.model.Chr9.601 |
| Clan72 | CYP709 | CYP709B | AT2G46960.2 | Clan72 | CYP734 | CYP734A | evm.model.Chr1.2564 |
| Clan72 | CYP709 | CYP709B | AT4G27710.2 | Clan72 | CYP734 | CYP734A | evm.model.Chr16.308 |
| Clan72 | CYP714 | CYP714A | AT5G24900.2 | Clan72 | CYP734 | CYP734A | evm.model.Chr21.1556 |
| Clan72 | CYP714 | CYP714A | AT5G24910.1 | Clan72 | CYP734 | CYP734A | evm.model.Chr3.389 |
| Clan72 | CYP715 | CYP715A | AT5G52400.1 | Clan72 | CYP735 | CYP735A | evm.model.Chr11.114 |
| Clan72 | CYP72 | CYP72A | AT3G14610.1 | Clan72 | CYP735 | CYP735A | evm.model.Chr8.427 |
| Clan72 | CYP72 | CYP72A | AT3G14620.1 | Clan72 | CYP749 | CYP749A | evm.model.Chr10.1457 |
| Clan72 | CYP72 | CYP72A | AT3G14630.1 | Clan72 | CYP749 | CYP749A | evm.model.Chr14.1949 |
| Clan72 | CYP72 | CYP72A | AT3G14640.1 | Clan72 | CYP749 | CYP749A | evm.model.Chr14.1950 |
| Clan72 | CYP72 | CYP72A | AT3G14650.1 | Clan72 | CYP749 | CYP749A | evm.model.Chr14.1951 |
| Clan72 | CYP72 | CYP72A | AT3G14660.2 | Clan72 | CYP749 | CYP749A | evm.model.Chr2.2183 |
| Clan72 | CYP72 | CYP72A | AT3G14680.1 | Clan72 | CYP749 | CYP749A | evm.model.Chr2.2197 |
| Clan72 | CYP72 | CYP72A | AT3G14690.2 | Clan74 | CYP74 | CYP74A | evm.model.Chr1.3000 |
| Clan72 | CYP72 | CYP72C | AT1G17060.1 | Clan74 | CYP74 | CYP74A | evm.model.Chr17.2212 |
| Clan72 | CYP721 | CYP721A | AT1G75130.1 | Clan74 | CYP74 | CYP74A | evm.model.Chr7.1491 |
| Clan72 | CYP734 | CYP734A | AT2G26710.1 | Clan74 | CYP74 | CYP74B | evm.model.Chr6.1178 |
| Clan72 | CYP735 | CYP735A | AT1G67110.1 | Clan85 | CYP707 | CYP707A | evm.model.Chr1.2955 |
| Clan72 | CYP735 | CYP735A | AT5G38450.1 | Clan85 | CYP707 | CYP707A | evm.model.Chr1.2956 |
| Clan74 | CYP74 | CYP74A | AT5G42650.1 | Clan85 | CYP707 | CYP707A | evm.model.Chr12.2029 |
| Clan74 | CYP74 | CYP74B | AT4G15440.1 | Clan85 | CYP707 | CYP707A | evm.model.Chr13.1982 |
| Clan85 | CYP702 | CYP702A | AT1G65670.1 | Clan85 | CYP707 | CYP707A | evm.model.Chr14.477 |
| Clan85 | CYP702 | CYP702A | AT3G30290.1 | Clan85 | CYP707 | CYP707A | evm.model.Chr14.512 |
| Clan85 | CYP702 | CYP702A | AT4G15300.1 | Clan85 | CYP707 | CYP707A | evm.model.Chr16.867 |
| Clan85 | CYP702 | CYP702A | AT4G15310.1 | Clan85 | CYP707 | CYP707A | evm.model.Chr18.490 |
| Clan85 | CYP702 | CYP702A | AT4G15393.1 | Clan85 | CYP707 | CYP707A | evm.model.Chr18.559 |
| Clan85 | CYP702 | CYP702A | AT4G15396.1 | Clan85 | CYP707 | CYP707A | evm.model.Chr2.3025 |
| Clan85 | CYP707 | CYP707A | AT2G29090.4 | Clan85 | CYP707 | CYP707A | evm.model.Chr3.1862 |
| Clan85 | CYP707 | CYP707A | AT3G19270.2 | Clan85 | CYP707 | CYP707A | evm.model.Chr7.1525 |
| Clan85 | CYP707 | CYP707A | AT4G19230.1 | Clan85 | CYP707 | CYP707A | evm.model.Chr9.696 |
| Clan85 | CYP707 | CYP707A | AT5G45340.1 | Clan85 | CYP716 | CYP716A | evm.model.Chr11.1348 |
| Clan85 | CYP708 | CYP708A | AT1G55940.2 | Clan85 | CYP716 | CYP716A | evm.model.Chr11.1351 |
| Clan85 | CYP708 | CYP708A | AT1G78490.2 | Clan85 | CYP716 | CYP716A | evm.model.Chr13.2522 |
| Clan85 | CYP708 | CYP708A | AT5G48000.5 | Clan85 | CYP716 | CYP716A | evm.model.Chr13.2523 |
| Clan85 | CYP716 | CYP716A | AT5G36110.2 | Clan85 | CYP716 | CYP716A | evm.model.Chr19.60 |
| Clan85 | CYP716 | CYP716A | AT5G36140.1 | Clan85 | CYP716 | CYP716A | evm.model.Chr3.1222 |
| Clan85 | CYP718 | CYP718 | AT2G42850.2 | Clan85 | CYP716 | CYP716A | evm.model.Chr3.2642 |
| Clan85 | CYP720 | CYP720A | AT1G73340.1 | Clan85 | CYP716 | CYP716A | evm.model.Chr5.2046 |
| Clan85 | CYP722 | CYP722A | AT1G19630.1 | Clan85 | CYP716 | CYP716A | evm.model.Chr5.2049 |
| Clan85 | CYP724 | CYP724A | AT5G14400.1 | Clan85 | CYP716 | CYP716A | evm.model.Chr6.128 |
| Clan85 | CYP85 | CYP85A | AT3G30180.1 | Clan85 | CYP716 | CYP716A | evm.model.Chr6.1344 |
| Clan85 | CYP85 | CYP85A | AT5G38970.3 | Clan85 | CYP716 | CYP716A | evm.model.Chr6.552 |
| Clan85 | CYP87 | CYP87A | AT1G12740.1 | Clan85 | CYP716 | CYP716A | evm.model.Chr6.553 |
| Clan85 | CYP88 | CYP88A | AT1G05160.1 | Clan85 | CYP716 | CYP716A | evm.model.Chr8.1620 |
| Clan85 | CYP88 | CYP88A | AT2G32440.2 | Clan85 | CYP716 | CYP716A | evm.model.Chr8.1624 |
| Clan85 | CYP90 | CYP90A | AT5G05690.2 | Clan85 | CYP716 | CYP716A | evm.model.Chr8.2344 |
| Clan85 | CYP90 | CYP90B | AT3G50660.2 | Clan85 | CYP716 | CYP716A | evm.model.Chr8.2350 |
| Clan85 | CYP90 | CYP90D | AT3G13730.1 | Clan85 | CYP716 | CYP716A | evm.model.Chr9.77 |
| Clan86 | CYP704 | CYP704A | AT2G44890.2 | Clan85 | CYP718 | CYP718 | evm.model.Chr1.430 |
| Clan86 | CYP704 | CYP704A | AT2G45510.1 | Clan85 | CYP720 | CYP720A | evm.model.Chr5.2903 |
| Clan86 | CYP704 | CYP704B | AT1G69500.1 | Clan85 | CYP722 | CYP722A | evm.model.Chr1.1518 |
| Clan86 | CYP86 | CYP86A | AT1G01600.1 | Clan85 | CYP722 | CYP722A | evm.model.Chr9.251 |
| Clan86 | CYP86 | CYP86A | AT1G63710.1 | Clan85 | CYP725 | CYP725A | evm.model.Chr1.429 |
| Clan86 | CYP86 | CYP86A | AT2G45970.1 | Clan85 | CYP85 | CYP85A | evm.model.Chr11.621 |
| Clan86 | CYP86 | CYP86A | AT4G00360.1 | Clan85 | CYP85 | CYP85A | evm.model.Chr14.1943 |
| Clan86 | CYP86 | CYP86A | AT5G58860.1 | Clan85 | CYP85 | CYP85A | evm.model.Chr8.752 |
| Clan86 | CYP86 | CYP86B | AT5G23190.1 | Clan85 | CYP87 | CYP87A | evm.model.Chr10.2171 |
| Clan86 | CYP86 | CYP86C | AT1G13140.1 | Clan85 | CYP87 | CYP87A | evm.model.Chr14.218 |
| Clan86 | CYP86 | CYP86C | AT1G13150.1 | Clan85 | CYP87 | CYP87A | evm.model.Chr5.1439 |
| Clan86 | CYP86 | CYP86C | AT1G24540.1 | Clan85 | CYP88 | CYP88A | evm.model.Chr1.947 |
| Clan86 | CYP86 | CYP86C | AT3G26125.1 | Clan85 | CYP88 | CYP88A | evm.model.Chr12.2265 |
| Clan86 | CYP94 | CYP94B | AT3G01900.1 | Clan85 | CYP88 | CYP88A | evm.model.Chr15.811 |
| Clan86 | CYP94 | CYP94B | AT3G48520.1 | Clan85 | CYP88 | CYP88A | evm.model.Chr21.1043 |
| Clan86 | CYP94 | CYP94B | AT5G63450.2 | Clan85 | CYP90 | CYP90A | evm.model.Chr4.489 |
| Clan86 | CYP94 | CYP94C | AT2G27690.1 | Clan85 | CYP90 | CYP90B | evm.model.Chr1.1538 |
| Clan86 | CYP94 | CYP94D | AT1G34540.1 | Clan85 | CYP90 | CYP90B | evm.model.Chr3.218 |
| Clan86 | CYP94 | CYP94D | AT3G56630.1 | Clan85 | CYP90 | CYP90B | evm.model.Chr7.1763 |
| Clan86 | CYP96 | CYP96A | AT1G47620.1 | Clan85 | CYP90 | CYP90D | evm.model.Chr1.1497 |
| Clan86 | CYP96 | CYP96A | AT1G57750.1 | Clan85 | CYP90 | CYP90D | evm.model.Chr1.2313 |
| Clan86 | CYP96 | CYP96A | AT1G65340.1 | Clan85 | CYP90 | CYP90D | evm.model.Chr7.2220 |
| Clan86 | CYP96 | CYP96A | AT1G66030.1 | Clan86 | CYP704 | CYP704A | evm.model.Chr4.304 |
| Clan86 | CYP96 | CYP96A | AT2G21910.1 | Clan86 | CYP704 | CYP704A | evm.model.Chr5.2570 |
| Clan86 | CYP96 | CYP96A | AT2G23180.1 | Clan86 | CYP704 | CYP704A | evm.model.Chr6.2598 |
| Clan86 | CYP96 | CYP96A | AT4G32170.1 | Clan86 | CYP77 | CYP704B | evm.model.Chr15.1863 |
| Clan86 | CYP96 | CYP96A | AT4G39480.1 | Clan86 | CYP77 | CYP704B | evm.model.Chr20.1607 |
| Clan86 | CYP96 | CYP96A | AT4G39490.1 | Clan86 | CYP86 | CYP86A | evm.model.Chr12.1338 |
| Clan86 | CYP96 | CYP96A | AT4G39500.2 | Clan86 | CYP86 | CYP86A | evm.model.Chr14.331 |
| Clan86 | CYP96 | CYP96A | AT4G39510.1 | Clan86 | CYP86 | CYP86A | evm.model.Chr17.1292 |
| Clan86 | CYP96 | CYP96A | AT5G02900.1 | Clan86 | CYP86 | CYP86A | evm.model.Chr17.1293 |
| Clan86 | CYP96 | CYP96A | AT5G52320.2 | Clan86 | CYP86 | CYP86A | evm.model.Chr19.593 |
| Clan97 | CYP97 | CYP97A | AT1G31800.1 | Clan86 | CYP86 | CYP86A | evm.model.Chr22.1326 |
| Clan97 | CYP97 | CYP97B | AT4G15110.1 | Clan86 | CYP86 | CYP86A | evm.model.Chr3.1036 |
| Clan97 | CYP97 | CYP97C | AT3G53130.1 | Clan86 | CYP86 | CYP86A | evm.model.Chr3.1627 |
|  |  |  |  | Clan86 | CYP86 | CYP86B | evm.model.Chr22.367 |
|  |  |  |  | Clan86 | CYP86 | CYP86B | evm.model.Chr4.1000 |
|  |  |  |  | Clan86 | CYP94 | CYP94A | evm.model.Chr4.1582 |
|  |  |  |  | Clan86 | CYP94 | CYP94B | evm.model.Chr1.1819 |
|  |  |  |  | Clan86 | CYP94 | CYP94B | evm.model.Chr7.2842 |
|  |  |  |  | Clan86 | CYP94 | CYP94B | evm.model.Chr9.1501 |
|  |  |  |  | Clan86 | CYP94 | CYP94C | evm.model.Chr10.2537 |
|  |  |  |  | Clan86 | CYP94 | CYP94C | evm.model.Chr15.324 |
|  |  |  |  | Clan86 | CYP94 | CYP94C | evm.model.Chr15.523 |
|  |  |  |  | Clan86 | CYP94 | CYP94C | evm.model.Chr15.526 |
|  |  |  |  | Clan86 | CYP94 | CYP94C | evm.model.Chr16.2035 |
|  |  |  |  | Clan86 | CYP94 | CYP94C | evm.model.Chr20.1814 |
|  |  |  |  | Clan86 | CYP94 | CYP94C | evm.model.Chr4.3072 |
|  |  |  |  | Clan86 | CYP94 | CYP94C | evm.model.Chr8.1792 |
|  |  |  |  | Clan86 | CYP94 | CYP94D | evm.model.Chr16.1391 |
|  |  |  |  | Clan86 | CYP94 | CYP94D | evm.model.Chr22.1631 |
|  |  |  |  | Clan86 | CYP94 | CYP94D | evm.model.Chr4.1963 |
|  |  |  |  | Clan86 | CYP96 | CYP96A | evm.model.Chr1.3671 |
|  |  |  |  | Clan86 | CYP96 | CYP96A | evm.model.Chr2.2196 |
|  |  |  |  | Clan86 | CYP96 | CYP96A | evm.model.Chr2.905 |
|  |  |  |  | Clan86 | CYP96 | CYP96A | evm.model.Chr2.906 |
|  |  |  |  | Clan86 | CYP96 | CYP96A | evm.model.Chr20.1609 |
|  |  |  |  | Clan86 | CYP96 | CYP96A | evm.model.Chr22.1702 |
|  |  |  |  | Clan86 | CYP96 | CYP96A | evm.model.Chr23.659 |
|  |  |  |  | Clan86 | CYP96 | CYP96A | evm.model.Chr6.1285 |
|  |  |  |  | Clan86 | CYP96 | CYP96A | evm.model.Chr7.776 |
|  |  |  |  | Clan86 | CYP96 | CYP96A | evm.model.Chr9.996 |
|  |  |  |  | Clan86 | CYP96 | CYP96A | evm.model.Chr9.998 |
|  |  |  |  | Clan97 | CYP97 | CYP97A | evm.model.Chr21.1228 |
|  |  |  |  | Clan97 | CYP97 | CYP97A | evm.model.Chr6.1948 |
|  |  |  |  | Clan97 | CYP97 | CYP97B | evm.model.Chr21.1310 |
|  |  |  |  | Clan97 | CYP97 | CYP97C | evm.model.Chr16.1836 |
